# Effective Connectivity during an Avoidance-Based Pavlovian-to-Instrumental Transfer Task

**DOI:** 10.3390/brainsci11111472

**Published:** 2021-11-06

**Authors:** Daniel J. Petrie, Sy-Miin Chow, Charles F. Geier

**Affiliations:** 1 Department of Human Development and Family Studies, Pennsylvania State University, University Park, PA 16802, USA; djp67@psu.edu (D.J.P.); quc16@psu.edu (S.-M.C.); 2 Social Science Research Institute, Pennsylvania State University, University Park, PA 16802, USA

**Keywords:** pavlovian-to-instrumental transfer, negative reinforcement, striatum, effective connectivity

## Abstract

Pavlovian-to-instrumental transfer (PIT) refers to a phenomenon whereby a classically conditioned stimulus (CS) impacts the motivational salience of instrumental behavior. We examined behavioral response patterns and functional magnetic resonance imaging (fMRI) based effective connectivity during an avoidance-based PIT task. Eleven participants (8 females; *M_age_* = 28.2, *SD* = 2.8, *range* = 25–32 years) completed the task. Effective connectivity between a priori brain regions engaged during the task was determined using hemodynamic response function group iterative multiple model estimation (HRF-GIMME). Participants exhibited behavior that was suggestive of specific PIT, a CS previously associated with a reinforcing outcome increased instrumental responding directed at the same outcome. We did not find evidence for general PIT; a CS did not significantly increase instrumental responding towards a different but related outcome. Using HRF-GIMME, we recovered effective connectivity maps among corticostriatal circuits engaged during the task. Group-level paths revealed directional effects from left putamen to right insula and from right putamen to right cingulate. Importantly, a direct effect of specific PIT stimuli on blood–oxygen-level-dependent (BOLD) activity in the left putamen was found. Results provide initial evidence of effective connectivity in key brain regions in an avoidance-based PIT task network. This study adds to the literature studying PIT effects in humans and employing GIMME models to understand how psychological phenomena are supported in the brain.

## 1. Introduction

Pavlovian-to-instrumental transfer (PIT) refers to a phenomenon whereby previously learned cues can influence goal-directed behavior [1,2]. PIT is thought to be an important factor in several health-related and cue-influenced behaviors, including food consumption and substance use [3,4,5,6]. Indeed, previous studies have argued that PIT may be a major factor why some individuals engage in drug-seeking behaviors (e.g., smoking) when presented with conditioned drug-related cues (e.g., viewing an ashtray) [7]. PIT established in the context of negative reinforcement has also been proposed as a model to better understand the nature of relapse, in that individuals respond (seek drugs) to remove aversive effects associated with abstinence [8,9].

In general, paradigms used to examine PIT are comprised of three phases. First, participants undergo instrumental conditioning, during which time they learn to associate specific behavioral responses (R) (e.g., button presses) with valued outcomes (O) (e.g., they learn R1-O1 and R2-O2 associations). Second, participants undergo Pavlovian (classical) conditioning where they learn, across repeated trials, to associate conditioned stimuli (CS) with outcomes (O) (e.g., CS1-O1, CS2-O2, CS3-O3). During a final test or ‘transfer’ phase, individuals are presented with previously learned Pavlovian cues in addition to similar but unconditioned stimuli (e.g., CS4 and CS5, two unconditioned control stimuli), as they engage in the learned instrumental behavior. Notably, this final phase occurs during extinction, when no incentives are given, so that instrumental behavior is not affected by their delivery. In other words, participants had the choice to utilize R1 and R2 for CS1-CS5 in the absence of any feedback (see Table 1 for contingencies of the PIT task). During the test phase, in humans and animal models, behavior is biased in that more frequent responding occurs on trials linked with previously learned cues (e.g., increased R1 for CS1, increased R2 for CS2) [10]. Of note, PIT effects have been demonstrated in experiments utilizing both positive and negative reinforcement. In the context of positive reinforcement, individuals demonstrate increase instrumental responding for an appetitive stimulus following Pavlovian learning trials [10]. In the context of negative reinforcement, individuals also increase instrumental responding but in this case to remove or avoid an aversive stimulus [2].

Previous research has described two different forms of PIT–specific and general [10,11,12]. In specific PIT, a conditioned stimulus (CS1) that was previously associated with a reinforcing outcome (O1) increases instrumental responding (R1) directed at the same outcome (O1). For example, if an individual usually smokes a cigarette after a certain meal, then consuming that meal in the future may lead to craving a cigarette. In general PIT, a CS increases instrumental responding towards a different, but often related, reinforcing outcome, even when the CS and the instrumental response never shared a reinforcing outcome. That is, nonselective increases in instrumental responding are observed–R1 and R2 would be increased for CS3. For example, smoking a cigarette after a meal could itself act as a cue that induces craving for another drug, such as alcohol.

Previous research indicates that dorsal striatum is involved in instrumental learning in specific and general PIT [13]. The dorsolateral striatum (e.g., putamen in humans) is associated with CS-R habitual learning [14]; where lesions of the dorsolateral striatum in rodents reduces both specific and general PIT. [15,16]. In contrast, the dorsomedial striatum (e.g., caudate in humans) is associated with R-O goal-directed learning [14,17]; where lesions of the dorsomedial striatum in rodents reduces specific PIT only [16]. Results from lesion studies in animals have been supported in human neuroimaging studies, which link dorsal striatum activity with the degree of instrumental responding, and specific and general PIT effects [8,18]. More specifically, research by Lewis and colleagues [8] found significant positive correlations among left putamen blood–oxygen-level-dependent (BOLD) responses during Pavlovian training and instrumental responding during the transfer phase for specific and general PIT stimuli. These findings support the conclusion that the putamen is a key region that mediates processes for instrumental learning during both specific and general PIT.

The insular cortex and cingulate cortex have also been implicated in PIT, but may be more specific to PIT during avoidance conditioning and learning [19]. For example, researchers have used functional magnetic resonance imaging (fMRI) during an avoidance conditioning task and found increased BOLD response in the insula and cingulate compared with neutral trials [20,21]. An fMRI meta-analysis corroborated these results by showing increased BOLD response in insula and cingulate during avoidance conditioning tasks [19]. Additionally, a version of the PIT task that uses instructed negative reinforcers, as opposed to the more studied primary and secondary reinforcers such as a foot shock or money, respectively, found increased activation during PIT transfer in bilateral insula and right cingulate [8]. In regards to connectivity with the dorsal striatum, an in vivo probabilistic tractography study mapped structural connectivity patterns between the insula and the putamen [22]. Additionally, tracing studies in nonhuman primates have also found corticostriatal projections from the cingulate to the putamen [23]. These findings suggests that the insula and cingulate are involved in avoidance-based PIT, and is corroborated by structural studies, which found projections to the putamen, forming an integrative network for PIT learning.

The studies discussed above, in particular the fMRI studies of PIT, have been primarily concerned with characterizing group-level localization maps using traditional general linear modeling (GLM)—to identify “which” brain regions are involved. To our knowledge, no studies to date have assessed effective connectivity patterns during PIT tasks in healthy human adults—or “how” brain regions are involved. As a first step toward addressing this issue, we applied hemodynamic response function group iterative multiple model estimation (HRF-GIMME) [24,25], a novel model-based approach which uses information at the group- and individual-level to construct effective connectivity maps, to further investigate between region connectivity during PIT. Importantly, HRF-GIMME models the direct and modulating effects of an event-related design fMRI task by incorporating person-specific HRF functions to account for the known variability in the shape of the HRF [26].

Although conclusions concerning group-level connectivity patterns are fundamental to advancing understanding of the brain regions that are implicated in PIT, the heterogeneity that exists in the patterns and strengths of brain connectivity in any given sample is equally critical and often overlooked [27,28]. Uninformed imposition of homogeneity assumptions in evaluating fMRI connectivity patterns may lead to erroneous conclusions that fail to characterize any of the individuals in the sample well [29,30]. One recent approach, group iterative multiple model estimation (GIMME) [31], aims to address the limitations of traditional functional connectivity analysis by using a dynamic network analysis approach that identifies group-level effective (i.e., directional) connectivity patterns from person-specific networks. Through multiple iterative evaluations of individual- and group-level effective connectivity maps, Gates and Molenaar (2012) demonstrated that GIMME far exceeded the performance of other effectivity connectivity methods, including Bayes net approaches, in recovering both the presence of a connection and the directionality of the connection from the benchmark simulated data sets generated by Smith et al., for comparison of fMRI network methods [32].

To perform iterative individual- and group-level model selection, GIMME utilizes at its core the extended unified structural equation model (euSEM) [25], which captures not only general effective connectivity patterns that are constant across conditions but also whether and how the effectivity connectivity patterns vary as influenced or modulated by external inputs, such as task conditions. The modulating effects on task-related inputs on effectivity connectivity patterns are also termed the “bilinear effects” [33]. In the present article, we utilized a new extension of GIMME, namely, GIMME with person-specific HRF (HRF-GIMME), which convolves HRFs to accommodate individual differences in responsivity to task-related inputs (e.g., delayed responses to task-related stimuli) [24,25,34,35]. HRF-GIMME can help identify in a more targeted way the brain regions that show sustained recruitment (i.e., showing continuity in dynamics over time) during PIT, the effective connectivity patterns of these regions, and whether and how such patterns differ under specific vs. general PIT, at both the group as well as individual levels.

Healthy adult participants performed an avoidance-based PIT task while simultaneously undergoing fMRI scanning. Time series data from select brain regions of interest (ROI) identified in Lewis et al. [8] during the test/transfer phase were extracted and modeled using HRF-GIMME. Our first aim was to replicate the behavioral findings as in previous studies of avoidance-based PIT [8,36], by assessing whether participants were engaging in the experiment correctly across the three stages of the task. During the instrumental learning phase, we aimed to validate that the participants learned the correct R-O pairings of O1 and O2. During the Pavlovian phase, we aimed to validate that the participants learned all five of the correct CS-O pairings. During the PIT phase, we aimed to replicate the findings from previous literature to assess whether participants exhibited specific and general PIT during extinction conditions when no outcomes were presented and were only shown CS. Our second aim was to extend GLM results reported in Lewis et al. [8] by demonstrating the use of HRF-GIMME in determining effective connectivity patterns among corticostriatal ROIs that are associated with specific and general PIT in humans. More specifically, we sought to characterize directional effective connectivity patterns from bilateral putamen to bilateral insula and right cingulate cortex due to their roles in negative reinforcement and PIT. Our third aim was to assess whether any direct or bilinear effects in relation to task demands were associated with PIT effective connectivity. For direct effects, we hypothesized that specific and general PIT stimuli would be associated with an increase in BOLD activity in the left putamen. For bilinear effects, we hypothesized that specific and general PIT would modulate the relationship among the left putamen and other selected ROIs based on previous tractography and tracing studies that suggest structural connectivity among these regions [22,23]. In other words, the strength of relations between left putamen and other ROIs would be influenced by whether or not specific and general PIT stimuli were being presented.

## 2. Materials and Methods

### 2.1. Participants

Upon receipt of approval by the local Institutional Review Board, 12 participants were recruited at a large university in the Northeast United States as part of a larger study assessing the interplay between goal-directed and habitual control. Consent for participation was obtained on the first visit to the laboratory. The final analysis included 11 participants (8 female; *M_age_* = 28.2, *SD* = 2.8, *range* = 25–32 years), as one participant was excluded due to failure to meet instrumental learning criteria (see procedure description). Of the 11 participants, 7 were White (64%), and 4 were Asian (36%). Inclusion criteria included: good general health, no diagnosed learning disabilities (e.g., ADHD), no diagnosed psychological condition that could impact comfort in the fMRI (e.g., anxiety), not on any medications known to influence body weight, taste, food intake, behavior, or blood flow, not claustrophobic, and not currently on a diet for weight loss. Exclusion criteria included: not within the age range (adults’ range 24–40 years), learning disability or other neurological or psychological conditions, type 1 or type 2 diabetes, and food allergies. Participants will also be excluded if they have any tattoos, permanent makeup, dental ware, pacemakers, or metal implants that would impede safe completion of the MRI.

We note that while the current study’s sample size (e.g., 11 participants) is low powered for traditional univariate fMRI analysis, GIMME and HRF-GIMME capitalize heavily on fitting network models at the individual level before finding prominent patterns at the group level. Initial work in fitting network models to single-subject multivariate time-series data found that dynamic factor models that estimate parameters from a block-Toeplitz matrix (i.e., GIMME), was able to yield acceptable parameter estimates with observations as low as 50 [37]. Thus, these approaches are powered by the length of each individual’s fMRI time series more so than the number of participants. Reliable results have been found using GIMME with simulated sample sizes as low as 10 participants and at least 100 data points [31]. For example, using simulated data from Smith et al. [32], the benchmark GIMME study found that for 10 participants, the presence precision (number of true connections divided by the number of total connections) was 89%, and the presence recall (number of true connections divided by the number of connection in simulation) was 99%. For the current study, each subject contributed a time series of 540 observations, therefore this analysis has reasonably high power to detect functional connectivity between ROIs at the individual level.

### 2.2. Procedure

A computer game paradigm was used to examine both specific and general PIT and was adapted from Lewis et al., [8]. At the beginning of the experiment, participants were told that they would be playing a computer game where their goal was to defend a fictional kingdom from attacking creatures (more details below). The PIT task has three phases: instrumental learning, Pavlovian conditioning, and a transfer test phase (phases are outlined in Table 1).

#### 2.2.1. Instrumental Phase

**Figure 1:**
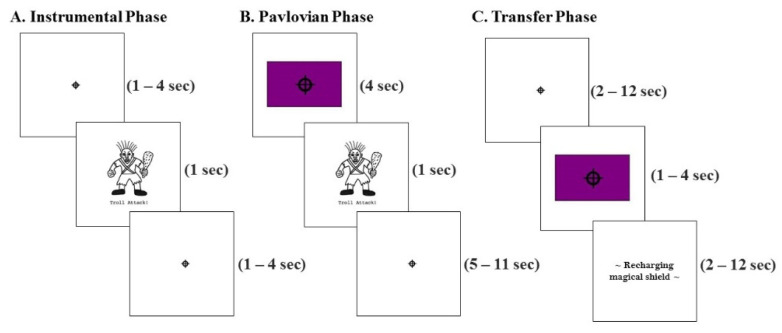
Schematic of the PIT task. (**A**) Instrumental phase: An aversive outcome (1 sec) occurred after each fixation cross. Participants were free to utilize R1 and R2. The correct response prolonged the onset of the next aversive event by 3 s. Participants completed two blocks of instrumental conditioning for each aversive outcome (O1 and O2). (**B**) Pavlovian Phase: participants viewed five randomly ordered CS-O contingencies and were told to remember the contingencies presented. (**C**) Transfer phase: Participants were shown CS1CS5 in random order after a fixation cross and before a “recharging” period. Participants were instructed to not respond during the recharge period but were free to respond at any other point during this phase.

During the instrumental phase, participants learned associations between two responses (R1 and R2), and the avoidance of two aversive outcomes (O1 and O2). Participants were initially instructed that they would be attacked by two (of three possible) creatures (e.g., goblin, troll, or ogre, counterbalanced across participants)—the aversive outcomes—and that they could use two available button presses that each yielded a different type of imaginary shield. Participants had to learn through trial and error which button press response would protect them from a specific type of attack (e.g., Button 1 yielded an imaginary shield that protected against goblin attacks). Participants completed two instrumental condition sessions where one session involved learning only R1-O1 contingencies, and the second session involved learning only R2-O2 contingencies. Sessions lasted 180 s each. During the sessions, the aversive creature was scheduled to appear in one second increments, unless the participant made the correct button press within this one second time period. If the correct button was pressed, the onset of the aversive creature was delayed by an additional three seconds. A fixation cross was presented on the screen at other times (see Figure 1A). At the end of the second session, participants were asked how effective each imaginary shield was at protecting against each aversive creature on a 1–10 scale (e.g., did R1 prevent O1 or O2 from happening?). For each aversive creature, the rating from the incorrect button press was subtracted from the rating of the correct button press. Participants with scores of ≤0 for either aversive creature were to be excluded from further analysis, because this would indicate that they did not learn the instrumental contingencies (e.g., R1-O1 and R2-O2). All participants were able to learn both instrumental contingencies. No imaging data were collected during this phase. Participants performed this task in the mock MRI scanner to approximate the MRI space and as a means to reduce the total amount of time each participant spent in the scanner, thus reducing participant motion effects and fatigue.

#### 2.2.2. Pavlovian Phase

In the Pavlovian phase, participants learned five stimulus–outcome (CS-O; conditioned stimulus–outcome) contingencies. Participants were told that a wizard would be teaching them about colored flags (CS) that represent what type of aversive creature (O) will be attacking. Participants were instructed to pay attention to what each flag represented because at the end of the phase the wizard would give them a quiz to see if they learned all 5 CS-O contingencies. For each trial, one of five CS-O pairings were presented such that each flag (CS1–CS5) was paired with either the previously trained aversive creatures (e.g., O1 and O2), a new aversive creature (O3), or one of two neutral outcomes (O4 and O5; O4 was a screen with a fixation cross, O5 was a screen that read “malfunction”). Each CS-O pairing was shown 9 times each for a total of 45 trials per session. The colored flags for each CS-O pairing were counterbalanced across participants. Colored flags appeared on the screen for four seconds. Then, the paired outcomes were presented for one second. A jittered inter-trial interval of either seven seconds, nine seconds, or eleven seconds separated each trial (Figure 1B). Participants were instructed to not make any button presses during this phase. At the end of the Pavlovian phase, participants were shown each colored flag and was asked by the wizard to respond verbally with the correct CS-O pairing. This was used as an exclusionary criterion in our data analysis, in order to ensure that participants learned all five CS-O pairings. One participant was excluded for not reporting all correct pairings. Thus, data from 11 participants were analyzed further.

#### 2.2.3. Transfer Phase

During the transfer phase, participants were instructed that the wizard would send out the five colored flags (CS1-CS5) that they had just learned, and that they could utilize the available shields (e.g., button presses) as they saw fit. The transfer phase was conducted under extinction conditions, no aversive creatures (i.e., no negative reinforcement) were shown during this phase. During the transfer phase, participants were instructed to freely respond with R1 and R2, or not at all, to the presentation of CS1–CS5. Each trial began with a fixation cross, then a conditioned stimulus (one of the colored flags) was presented on the screen for four seconds. Following the stimulus presentation, a jittered 2–12 s screen that said “recharging magical shield” was shown (Figure 1C). Participants were instructed not to respond when the “recharging magical shield” screen was displayed. However, participants were free to respond, or to not respond, during the pre-stimulus fixation period and when the stimuli were being presented. The pre-stimulus period was included in order to assess baseline responding. Each stimulus (CS1–CS5) was shown 12 times in random order for a total of 60 trials.

### 2.3. Behavioral Analysis

As in previous behavioral analyses using this task [8,36], we assessed specific and general PIT by comparing instrumental responses (R1 and R2) across the five types of stimuli (CS1–CS5) and compared with the pre-stimulus fixation period. Thus, a specific PIT effect was defined as increase in participants’ responses toward the stimuli that were initially trained (e.g., if presented with CS1, participants will respond with R1; if presented with CS2, participants will respond with R2). A general PIT effect was defined as a non-selective increase in responding for the stimuli that was not trained on during the instrumental phase (e.g., if presented with CS3, participants will on average equally press R1 and R2). All post-hoc tests within a family of comparisons were corrected for multiple comparisons using the Bonferroni correction [38].

### 2.4. fMRI Acquisition

Images were acquired using a 3T Siemens MAGNETOM Prisma Fit Scanner with a 20-channel head coil at the Social, Life, and Engineering Sciences Imaging Center (SLEIC). Structural images were collected using a T1-weighted magnetization-prepared rapid acquisition gradient echo (MPRAGE) sequence to acquire 192 slices (0.9 × 0.9 × 0.9 mm voxels). Functional images were collected using a T2*-weighted gradient single-shot blood-oxygen-level-dependent (BOLD) echo planar imaging (EPI) sequence to acquire 33 slices (3 × 3 × 4 mm voxels, TR = 2 s, TE = 25 ms, flip angle = 70°, FoV = 240 × 240, slice gap = 0 mm). Stimuli were generated using E-prime (Psychology Software Tools, Pittsburgh, PA) and projected onto a screen positioned behind the magnet. Participants viewed the screen via a mirror attached to the head coil. Functional images were acquired during the Pavlovian phase and the transfer phase. 

### 2.5. fMRI Preprocessing

Functional images were preprocessed using standard steps in AFNI [39]. First, images were corrected for slice timing effects. Next, translational and rotational head motion estimates were calculated and all images were aligned to the minimum outlier volume using a cost function (lpc + ZZ). Volumes for which translational movements exceeded 0.3 mm relative to the previous volume, as well as TRs with outlier intensity fractions greater than 5%, were identified and later censored from deconvolution analysis. Functional and structural images were then nonlinearly warped into standard space (MNI152_2009_template; Montreal Neurological Institute). Functional images were smoothed with a Gaussian filter set at 4.0 mm full-width at half maximum. Each voxel’s time series were scaled to a mean of 100 for evaluation as percent signal change. Deconvolution analysis followed, using AFNI’s 3dDeconvolve. Regressors of no interest included motion estimates (translational and rotational) and their derivatives as well as a fourth-order polynomial function to remove low frequency scanner drift during the runs. No task specific regressors were included; rather, the residual time series after deconvolution from each participant were carried forward for further analysis.

### 2.6. ROI Selection and Time Series Processing

Our aim was to assess functional relationships among brain regions known to be involved in specific and general PIT. As such, we examined five regions of interest (ROIs), including bilateral putamen, bilateral insula, and right cingulate based on previous neuroimaging work with the PIT task in a aversive context using negative reinforcement [8] (Table 2). As these regions were reported in Talairach space by Lewis et al. [8], we used online tools to convert to MNI space (http://sprout022.sprout.yale.edu/mni2tal/mni2tal.html, accessed on 29 October 2021). 

For each of the 5 ROIs, a 10 mm diameter sphere mask was generated in AFNI, centered on the coordinates in Table 2. One mean time series per ROI, per subject was computed by averaging the signals from each voxel in the residual time series map covered by the sphere mask, using AFNI’s 3dmaskave tool. Time series from each ROI were concatenated across participants to produce a single matrix with 5 columns (one per ROI) and 540 rows (11 participants, each contributing 540 time points). This matrix was used as input for analysis in HRF-GIMME (see below). In order to assess how connectivity patterns may be affected in the presence of task stimuli, we extracted from E-prime a vector of stimulus onset and duration times using AFNI’s timing_tool.py. We then combined the vector of stimulus onset times for CS1 and CS2 to reflect specific PIT. Finally, we appended the stimulus onset times for specific and general PIT to the time series data for each participant for analysis. Thus, each participant produced a single matrix with seven columns (five for each ROI, one for specific PIT, one for general PIT) and 540 rows (11 participants, each contributing 540 time points).

### 2.7. HRF-GIMME

As in traditional GIMME, HRF-GIMME assumes, at its core, that individuals’ fMRI time series can be described using the euSEM model [25]. For continuity, the notation used by Duffy and colleagues [24] was retained for the present model expressed as:

(1) $$y_{i,t}=\left(A_{i}^{i}+A^{g}\right)y_{i,t}+\left(\phi_{i}^{i}+\phi^{g}\right)y_{i,t-1}+\left(\gamma_{i}^{i}+\gamma^{g}\right)u_{i,t}+\left(\tau_{i}^{i}+\tau^{g}\right)u_{i,t}y_{i,t}+\zeta_{t\prime }$$

Where $y_{i,t}$ ndicates the ROI time series for an individual *i* at time *t*, *u_i,t_* indicates a bivariate binary time series marking the presentation of specific and general PIT stimuli convolved with the HRF for an individual *i* at time *t* [34]. All elements in Equation (1) are separated by person (*i*) and group (*g*) superscripts to distinguish between effects that exist at the individual and the group level, respectively. In particular, *A* indicates the contemporaneous relationships among ROIs, ϕ indicates the lagged auto-regression and cross-regression (effective connectivity) coefficients among the ROIs, γ indicates the effects of the input series, τ indicates the bilinear effects, namely, the extent to which the contemporaneous associations among the ROIs vary by the bivariate binary input time series, and ζ indicates dynamic errors that are assumed to be a white noise process with zero means and diagonal covariance matrix. Directionality or effective connectivity in GIMME is estimated by assessing whether a given ROIs time series can predict another ROIs time series (either contemporaneously or lagged) after controlling for autoregressive effects. Controlling for autoregressive effects is necessary for establish Granger causality, where an ROI predicts activity in another ROI above and beyond the extent to which the ROI predicts itself over time [40]. GIMME is freely available through the R platform [41] (https://CRAN.R-project.org/package=gimme; version 0.7-3; R version 4.0.2, accessed on 29 October 2021).

For the purpose of this study, we focused on comparing group- and individual patterns of contemporaneous connections, even though lagged effects were also included in the model. Modeling lagged effects ensures unbiased estimations of contemporaneous connections, as suggested by simulations that show that not accounting for lagged effects can lead to spurious contemporaneous results [42]. However, successful detection of coherent group patterns of lagged connections is contingent on the time-intervals between successive measurements, and other considerations [43]. Because the time resolution of fMRI data is notably coarser than the millisecond scale of actual neural events [44], systematic patterns of group and individual difference in patterns are typically more saliently reflected in the contemporaneous connections.

As distinct from traditional GIMME, HRF-GIMME models task-related effects in Equation (1) using individual-specific HRFs. HRF-GIMME works under the assumption that the shape of the HRF varies more between people than within people [26]. Thus, HRF-GIMME derives person-specific HRF parameters that are estimated via a smoothed finite impulse response [45]. The smoothed finite impulse response makes no assumption about the shape of the HRF and has been found in past research to recover the true shape of the HRF [46].

HRF-GIMME estimates functional connectivity maps via a data-driven forward selection process of model building and then model pruning. First, individual connectivity maps are estimated and used to derive a functional connectivity map that characterizes the majority of the sample. This is performed using Lagrange multiplier tests (i.e., modification indices) [47]. Lagrange multiplier tests indicate the extent to which a given path, if added to the model, would significantly improve model fit. Group paths are retained if they improve model fit for 75% of the sample. This search-and-add procedure continues until there are no additional paths that improve model fit. Next, if any paths do not reach significance, they are pruned from the group level network model. This search-and-add procedure is then repeated at the individual level. That is, for each individual in the sample, the final group-level model is fit, then Lagrange multiplier tests are used to determine whether adding additional paths to the individual model would improve model fit. Excellent fit, as described by Gates and Molenaar [31], is obtained if two out of four commonly used fit indices thresholds are met: comparative fit indices (CFI) >0.95; non-normed fit index (NNFI) >0.95; root mean squared error of approximation (RMSEA) <0.05; standardized root mean residual (SRMR) <0.05. In sum, GIMME offers the ability to extract a homogenous network structure that characterizes most individuals (in our case, 75% of the sample) at the group-level, but allows researchers to assess person-specific heterogeneity through the individual network structures, which helps describe variability in psychological processes that are present between individuals and across time [29].

HRF-GIMME is currently the only approach that estimates the effects of stimuli on neural activity while also estimating contemporaneous and lagged relationships among ROIs. We utilized HRF-GIMME to estimate effective connectivity maps for specific and general PIT stimuli to assess whether differing patterns of connectivity emerged when engaging in the task. More specifically, we expected effective connectivity patterns from bilateral putamen to bilateral insula and right cingulate at the group level. This was tested by evaluating the presence of significant cross-regression pathways among these ROIs in the majority (>75%) of the participants under conditions with general and specific PIT. Additionally, we expected both direct and bilinear effects of specific and general PIT on left putamen, a key brain region implicated in PIT.

## 3. Results

### 3.1. Aim 1: Replication of Behavioral Findings

Assessing task performance occurred in three phases, where the goal was to assess whether participants learned the correct R-O and CS-O pairings (instrumental conditioning phase and Pavlovian conditioning phase), and to replicate the behavioral findings from Lewis et al. [8] during the PIT phase; which found behavioral evidence for specific and general PIT.

#### 3.1.1. Instrumental Phase

**Figure 2:**
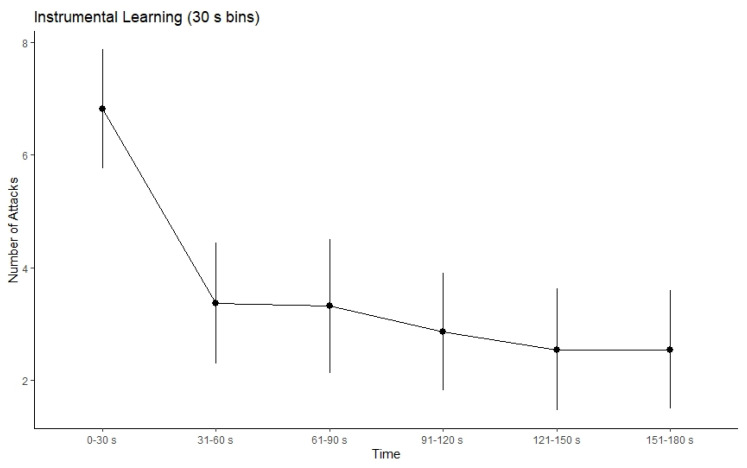
Number of attacks (30 s bins) during the instrumental phase. There was a significant decrease in the number of attacks from the first 30 s bin to the last 30 s bin (*p* < 0.05), suggesting that participants learned the correct R-O contingencies. Error bars represent standard error of the mean.

To measure instrumental learning, we separated each 180-s block into six 30-s bins in order to compare the total number of attacks from the first 30-s bin to the last 30-s bin (Figure 2). This approach has been used in past literature to determine successful avoidance learning [8,48,49,50]. The average number of attacks in the first 30-s bin was 6.82 attacks (*range* = 0–14, *SD* = 4.95, *SE* = 1.06). The average number of attacks in the last 30-s bin was 2.55 attacks (*range* = 0–14, *SD* = 4.91, *SE* = 1.05). We found a significant decrease in the amount of aversive outcome attacks from the first 30-s bin to the last 30-s bin (*t*_10_ = 5.55, *p* < 0.05). Additionally, this decrease in experienced aversive outcomes happened regardless of the creature type (O1: *t*_10_ = 3.04, *p* < 0.05; O2: *t*_10_ = 3.83, *p* < 0.05) suggesting that both R-O contingencies were learned during this phase. After the completion of the instrumental phase, all participants were verbally asked to report how effective each response was at preventing each outcome on a scale of 1 (least effective) to 10 (most effective). All participants were able to correctly pair each response to each outcome verbally. Verbal ratings were as followed: R1-O1 (correct pairing), mean = 8.73, *SD* = 1.56; R1-O2 (incorrect pairing), mean = 1.55, *SD* = 0.93; R2-O2 (correct pairing), mean = 9.09, *SD* = 1.30; R2-O1 (incorrect pairing), mean = 1.36, *SD* = 0.81.

#### 3.1.2. Pavlovian Phase

Following the Pavlovian phase, only one participant was unable to verbalize the outcomes associated with CS1-CS5 by answering, for each cue, the question “What did this signal represent?”, and therefore, was removed from further analysis. All other participants correctly learned all five CS-O contingencies.

#### 3.1.3. Transfer Phase

**Figure 3:**
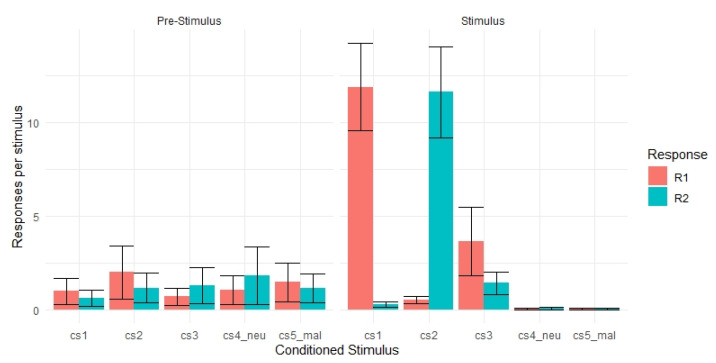
Number of responses per stimulus, by trial type, during the transfer phase. Specific transfer was observed; CS1 was associated with increased instrumental responding of R1 compared to R2 and compared to the pre-stimulus period. CS2 was associated with increased instrumental responding of R2 compared to R1 and compared to the pre-stimulus period (all *p* < 0.05). We did not observe a general transfer effect. CS3 did not increase R1 or R2 responding compared to the pre-stimulus period (all *p* > 0.05). Error bars represent standard error of the mean.

In line with previous studies [8,36], we measured specific and general PIT by comparing instrumental responding across all five stimulus types and during stimulus presentation compared to pre-stimulus presentation (Figure 3). Each CS was shown 12 times for a total of 60 trials. In order to compare instrumental responding among all five stimulus types, we divided the amount of instrumental responding by 12, the number of times each stimuli was shown, to reflect the average amount of responding per stimulus. The range of responses per stimulus were 0–22.5 (mean = 2.10, *SD* = 2.75, *SEM* = 0.83). For a full table of average responses per stimulus across the pre-stimulus and stimulus period please see Supplementary Table S1. A three-way repeated-measures ANOVA probing the effects of interval (pre-stimulus presentation and stimulus presentation), stimuli (CS1-CS5), and response (R1 and R2), revealed a significant main effect of stimulus (*F*_2, 20.01_ = 15.18; *p* < 0.05; *η*^2^ *_g_* = 0.13). Additionally, we observed a significant stimulus × response interaction (*F*_1.67, 16.73_ = 12.19; *p* < 0.05; *η*^2^ *_g_* = 0.21), and a significant stimulus × interval interaction (*F*_1.90, 19.01_ = 19.42, *p* < 0.05; *η*^2^ *_g_* = 0.14). A three-way stimulus × interval × response interaction was also observed (*F*_1.89, 18.86_ = 15.43, *p* < 0.05; *η*^2^ *_g_* = 0.22). For the full results of the three-way ANOVA please see Supplementary Table S2.

The significant three-way interaction was further analyzed via multiple pairwise comparisons to probe the effects of interval (pre-stimulus presentation and stimulus presentation) and response (R1 and R2) for each conditioned stimulus (CS1–CS5). For each comparison, the Bonferroni adjustment was applied. Consistent with our hypothesis that specific PIT stimuli (CS1 and CS2) would selectively increase instrumental responding of R1 and R2 respectively compared to the pre-stimulus period, we expected and found a significant increase in R1 instrumental responding for CS1 compared to the pre-stimulus period (*t*_10_ = −4.00, *p* < 0.05), and a significant increase in R2 responding for CS2 compared to the pre-stimulus period (*t*_10_ = −3.81, *p* < 0.05). All other pairwise comparisons were non-significant. Therefore, participant’s behavior was suggestive of specific PIT, participants displayed a selective increase in responding when the CS and the R shared a Pavlovian outcome. We did not observe a significant general PIT effect, participants did not display a non-selective increase in responding when the CS and the R never shared a Pavlovian outcome. Thus, we were able to partially replicate the behavioral findings from Lewis and colleagues [8]. For full results of the multiple pairwise comparisons please see Supplementary Table S3. 

### 3.2. Aim 2: HRF-GIMME Results

**Figure 4:**
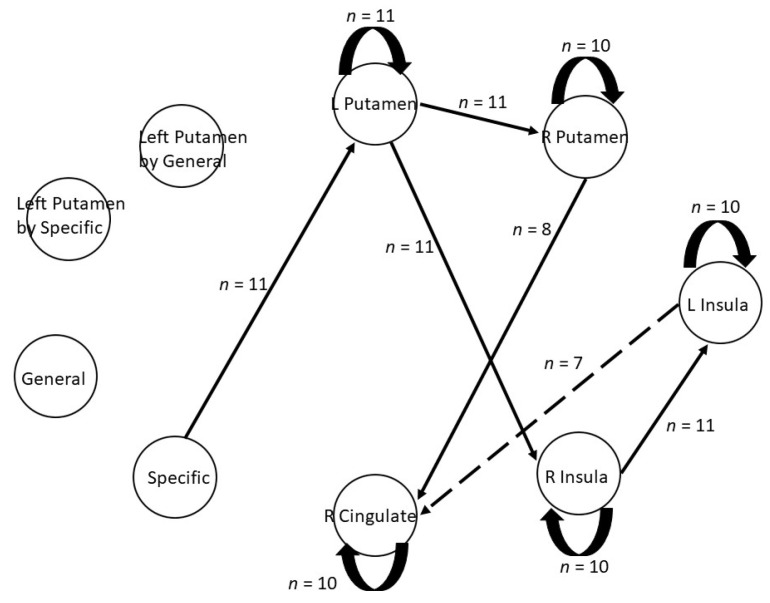
Group-level effective connectivity map recovered by HRF-GIMME. Solid lines represent contemporaneous connections; dotted lines represent lagged connections, curved black arrows represent autoregressive effects. Note that variables specific and general indicates the direct effects of the task on the other ROIs. In other words, the degree to which a task influences variability in BOLD activity at the corresponding ROI. *n* = number of participants in the sample that had a significant path at the group-level, R = right, L = left.

Figure 4 shows the group-level effective connectivity map during PIT. The resulting network fits the data well for all participants with average fit indices of: RMSEA = 0.07, SRMR = 0.04, CFI = 0.96, and NNFI = 0.91 (for a list of fit statistics of all participants please see Table 3). All models contain 11 group-level paths (thick black lines): five were autoregressive paths within each ROI that was estimated in the null model (black circular arrows), four are contemporaneous paths (solid black arrows), and one was a lagged path (dashed black arrows). Consistent with our hypothesis that directional connectivity patterns will emerge from bilateral putamen to bilateral insula and right cingulate cortex due to their roles in negative reinforcement and PIT, we found a significant group-level contemporaneous paths between left putamen and right insula, and a significant ipsilateral path between right putamen and right cingulate. Additionally, HRF-GIMME recovered significant contemporaneous paths between bilateral putamen and bilateral insula. HRF-GIMME also recovered a lagged group-level path from left insula to right cingulate. These group-level paths are estimated across all participant, with each participant having a unique estimate.

**Figure 5:**
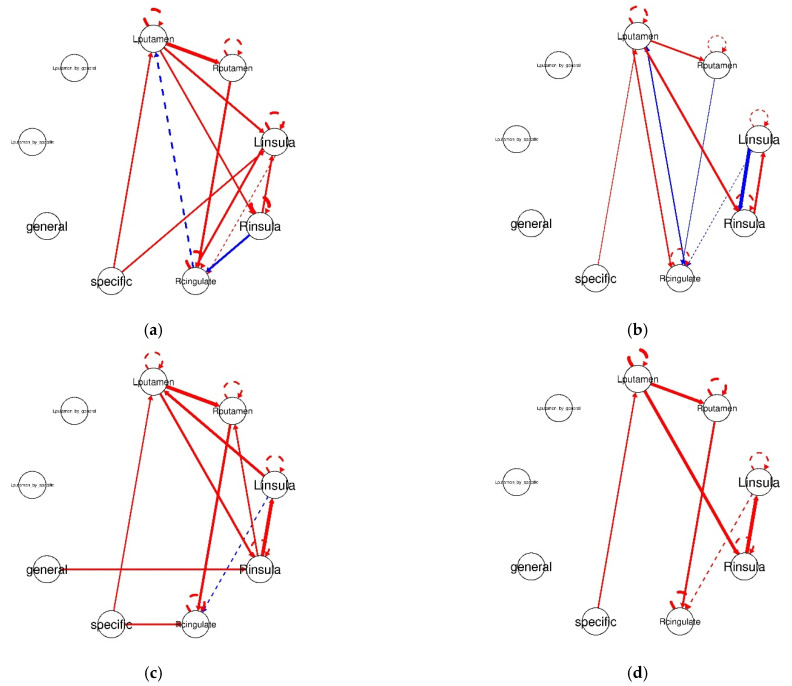
Effective connectivity plots recovered by HRF-GIMME for four participants. Red lines indicate a positive connection, blue lines indicate a negative connection, solid lines indicate contemporaneous connections, and dashed lines indicate a lagged connection of the first order. The thickness of the lines indicates the magnitude of the connection, such that thicker lines indicate a stronger magnitude of connectivity. Note that variables specific, and general indicates the direct effects of the task on the other ROIs. In other words, the degree to which a task influences variability in BOLD activity at the corresponding ROI. (**a**) Participant 1′s effective connectivity plot. (**b**) Participant 4′s effective connectivity plot. (**c**) Participant 5′s effective connectivity plot. (**d**) Participant 9′s effective connectivity plot.

To highlight the heterogeneity in the sample during PIT, we present four connectivity maps that were retained via HRF-GIMME in Figure 5 (please see Supplementary Figures S1–S11 for connectivity maps for all participants). Participant 1 had the highest number of individual-level paths (Figure 5a). Participant 1 had an additional direct effect of specific PIT on the BOLD response in the left insula. Participant 1 also displayed increased corticostriatal connectivity patterns from the left putamen and the left insula. Participant 4 had the highest number of negatively weighted paths, and a relatively weak (but significant) direct effect of specific PIT on left putamen (Figure 5b). For example, the β weight for Participant 1′s direct connection of specific PIT on left putamen was 0.18 compared to Participant 4′s β weight of 0.09. Participant 5 was the only participant who had three direct effects of PIT stimuli (Figure 5c). For specific PIT, Participant 5 had direct paths to the left putamen at the group-level and right cingulate at the individual-level. Additionally, Participant 5 had a direct general PIT path to the right insula. Finally, we choose to highlight Participant 9 due to HRF-GIMME only estimating group-level paths and no unique individual-level paths (Figure 5d). Of note, Participant 9 only displayed positive paths (full results from the HRF-GIMME analysis, including beta weight estimates for all paths for all individuals can be found on our OSF page https://osf.io/ykcr7/, last accessed on 8 October 2021).

### 3.3. Aim 3: Assessing Direct and Bilinear Effects from HRF-GIMME

In line with our hypothesis, a direct path was recovered at the group-level between the specific PIT stimuli and left putamen. In other words, the direct effect of specific PIT influences the participants’ BOLD activity in the left putamen. Contrary to our hypothesis, we did not observe a direct effect of general PIT on BOLD activity in the left putamen. However, at the individual-level, one participant had a direct effect of specific PIT on BOLD activity in the left insula and one participant had a direct effect of specific PIT on BOLD activity in the right cingulate. Additionally, one participant had a direct effect of the general PIT stimulus on BOLD activity in the right insula. Finally, contrary to our hypothesis, we did not observe any bilinear effects of specific or general PIT between left putamen and our chosen ROIs.

## 4. Discussion

Our aims in the current study were threefold. First, we aimed to reproduce behavioral effects related to specific and general PIT as reported in Lewis et al. [8] in an independent sample of participants. Our results partially replicated prior work [8,36]. Participants were able to learn the correct R-O contingencies that are indicative of instrumental learning. Additionally, participants were able to learn the correct S-O contingencies that are indicative of Pavlovian learning. Importantly, we found evidence suggesting specific PIT during the transfer phase; participants displayed a selective increase in instrumental responding when the CS and the R shared a Pavlovian outcome (Figure 3). However, we did not observe a significant general PIT effect in our sample. This null finding most likely stems from our small sample size, and future work with a larger sample may be able to replicate past results using this task. Other possibilities also warrant consideration for the null effect during general PIT. For example, it could be the case that the general motivational aspects of the aversive stimuli in this task (e.g., O1 and O2 responses are congruent with O3 responses) might not be strong enough to induce general PIT effects. It may be the case that the motivational salience of the instructed threat involves a higher degree of commitment and imagination from participants (as opposed to primary or secondary threats) making it generally more challenging to observe general PIT effects across individuals. Additionally, it should be noted that other research using avoidance-based PIT tasks have not observed general transfer effects [51]; although this work was conducted in participants with obsessive-compulsive traits and used differing avoidance stimuli. In sum, more research with a larger sample size and perhaps further investigation into the roles of motivational salience attributed to task reinforcers is needed to understand the behavioral mechanisms that support both specific and general PIT effects. 

Our second aim was to extend the group-level GLM results reported in Lewis et al., [8] by determining, for the first time, effective connectivity patterns among select corticostriatal ROIs associated with avoidance-based PIT task performance using HRF-GIMME [24,25]. More specifically, we sought to characterize directional effective connectivity among bilateral putamen, bilateral insula, and right cingulate cortex; these regions were selected due to their putative roles in negative reinforcement and PIT. Broadly, our findings support previous studies indicating that corticostriatal circuits are engaged during PIT in healthy humans [8,18,52,53]. Our results extend the literature by providing initial data related to information flow in a PIT task network. That is, we identified group-level effective connectivity paths that project from the left and right putamen to the right insula and right cingulate, respectively. Note that the presence of group-level connections among these regions suggests valid detection by HRF-GIMME at the 75% group cutoff. These group-level connections may serve as initial clues as to the flow of information through corticostriatal circuits during avoidance-based PIT in healthy human adults. We also provide initial evidence that supports the notion that considerable heterogeneity exists across individuals in effective connectivity patterns [27,28]. Utilizing HRF-GIMME allowed us to balance discovering generalizable findings from group-level connection that likely exists for all individuals as well as discovering individual-level differences in effective connectivity maps. The traditional GLM approaches used in fMRI analysis aggregates information across all individuals to arrive at a group-level connectivity map that may identify spurious paths that do not describe any individual in the sample [29]. HRF-GIMME is a complementary approach to traditional fMRI analysis because it allows for individual variability in connectivity patterns while not aggregating across individuals by averaging. GIMME first estimates group-level patterns which represents generalizable connections that likely exist for the sample. Then, for each individual, the model search builds on the estimated group-level paths to find individual-specific connections. In the current study, we show the value of HRF-GIMME in studying PIT by showing group-level corticostriatal paths, while allowing individual-path estimates that highlight the heterogeneity in effective connectivity patterns.

The heterogeneity in effective connectivity patterns during PIT complements recent findings using other neuroimaging methods such as diffusion tensor imaging (DTI). These studies found strong connectivity between putamen and premotor cortex [54,55,56], however, more recent work has found that individual differences in this connection did not predict the strength of PIT [57]. Recent studies have suggested that DTI research should take into account the influence of spatial systematic errors that may have been introduce due to a non-uniform magnetic field gradient [58,59]. One method that could account for the non-uniform magnetic field gradient is by utilizing the B-matrix spatial distribution in DTI (BSD-DTI) technique [60,61]. Efforts in accounting for systematic errors from non-uniform magnetic field gradients will help better characterize anatomical connectivity patterns among PIT regions. In sum, future work should aim to assess if other connections can explain variability in performance during PIT, especially in avoidance-based contexts, by employing a multimodal approach, where researchers utilize techniques that assess structural, functional, and effective connectivity. This future direction can help researchers to better elucidate psychological phenomena such as PIT and also better characterize the large amounts of heterogeneity seen in these processes.

Our third aim was to assess whether any direct or bilinear effects in relation to task demands were associated with PIT effective connectivity. In the current study, we found a direct effect of specific PIT stimuli on BOLD activity in the left putamen. These results complement the animal and human neuroimaging literature by again showing the involvement of the putamen during specific PIT. We did not find any evidence for bilinear effects at the group-level, suggesting that specific and general PIT stimuli are not modulating connectivity patterns between left putamen and the other selected ROIs during the task. This may be due to the nature of the PIT paradigm used in the current study. Recent research has suggested that HRF-GIMME can more reliably detect task related effects with slow-event related designs where the inter-trial interval (ITI) is approximately as long as the HRF [24]. In the current study, our ITIs were jittered and ranged from 3–16 s. It could be the case that heterogeneity in the sample made it more difficult to detect signal-contingent differences in connectivity patterns because the signal was weaker across some individuals. The lack of group-level bilinear task effects on the effective connectivity maps matches recent research using HRF-GIMME, where high levels of heterogeneity between individuals made detecting task-related effects more difficult at the traditional cutoff of 75% [24]. Additionally, Duffy and colleagues [24] suggest that task designs that evoke weaker effects may be missed when using HRF-GIMME to model rapid event-related designs. The combination of the rapid event-related design and the nature of HRF function results in less variability in the expected HRF making the detection of bilinear effect more difficult at the 75% cutoff value. The authors suggest lowering the cutoff threshold from 75% to 51% in order to increase the power to detect effects from task-related variables. We chose to keep the 75% cutoff due to the small sample size, and that this is the first-time effective connectivity maps have been estimated for an avoidance-based PIT task. Future research should aim to have a larger sample size in order to justify lowering the cutoff to 51%.

Our findings should be considered in the context of several limitations. First, the sample size is too small to be considered representative of a population. GIMME has performed well with as low as 10 participants [31] given that GIMME estimation is dependent on the length of the time series rather than the number of participants. However, the ability to conduct post hoc tests using GIMME output is limited due to the small sample size. Additionally, relating performance during the task to heterogeneity in network connectivity in a formal way would require methodological developments within the GIMME framework. Future work will expand this sample and assess whether behavioral performance during the PIT task is associated with effective connectivity parameters that are estimated from GIMME. Another potential limitation is the number of brain ROIs to assess when using in GIMME. While there is no formal limit to the number of selected ROIs, a maximum of 25 is suggested by the GIMME developers [31]. Other methods ought to be used if a whole brain (or whole network) approach is desired. Additionally, another limitation has to do with whether these transfer effects rely on the same brain regions across avoidance-based PIT tasks. This potential limitation stems from the differing learning mechanisms that are invoked across these tasks. During Pavlovian and instrumental learning S-O or R-O associations are either acquired via direct experience (e.g., learning to avoid a shock by pressing a button), or instructed about the relationship between the cue and the response (e.g., being instructed to remove an image of a creature by pressing buttons). Learning via experience or via instruction may share similar neural recruitment, but learning by instruction taxes additional cognitive mechanisms [62], and as such, may differentially effect the circuits that underlie avoidance based PIT. In sum, the current research on avoidance-based PIT in humans is emerging [63], but a paucity of extant studies and the inability to directly compare them makes assessing the neural underpinning of PIT challenging. An interesting future direction would be to examine individual-level differences in transfer effects, and assess whether these differences in PIT effects are associated with consummatory or drug seeking behavior.

## 5. Conclusions

In summary, this study is the first to our knowledge to assess behavior and effective connectivity during an avoidance-based PIT task using HRF-GIMME. Behaviorally, we found evidence for specific PIT; participants increase instrumental responding for a conditioned stimulus that previously shared a Pavlovian outcome. However, we did not find evidence for a general PIT effect. Effective connectivity results complement past research suggesting that corticostriatal circuits are recruited during the PIT task and add new information related to possible directional information flow through a PIT network. Importantly, we also found a direct effect of specific PIT stimuli on left putamen activity. Results from this study should be extended to more fully characterize behavior and neural underpinnings of PIT such that we main gain insight into important health behaviors (e.g., eating, drug use) that are affected by PIT. 

## Figures and Tables

**Table 1:** Contingencies present in PIT task.

Instrumental Phase	Pavlovian Phase	Transfer Phase
R1–O1	CS1–O1	CS1: R1 vs. R2
R2–O2	CS2–O2	CS2: R1 vs. R2
	CS3–O3	CS3: R1 vs. R2
	CS4–O4	CS4: R1 vs. R2
	CS5–O5	CS5: R1 vs. R2

Note. R = response, O = outcome, CS = conditioned stimulus.

**Table 2:** Regions of interest used in this study. All coordinates are in MNI space.

Region of Interest	Hemisphere	x	y	z
Putamen	L	−23	7	4
Putamen	R	18	10	0
Insula	L	−42	6	0
Insula	R	37	4	3
Cingulate	R	2	11	46

Note. L = left, R = right.

**Table 3:** Fit statistics.

Participant	RMSEA	SRMR	CFI	NNFI
1	0.081	0.0491	0.9523	0.8886
2	0.0736	0.0344	0.9552	0.9051
3	0.0614	0.0453	0.9553	0.8984
4	0.0871	0.037	0.96	0.9112
5	0.0679	0.045	0.9699	0.9315
6	0.0705	0.0491	0.9668	0.9244
7	0.0765	0.0408	0.957	0.9045
8	0.0778	0.0377	0.9572	0.9049
9	0.0548	0.0229	0.9843	0.9676
10	0.0975	0.0454	0.9513	0.8984
11	0.0681	0.0411	0.9659	0.9261

Note. RMSEA = root mean squared error of approximation; SRMR = standardized root mean square residual; CFI = comparative fit index; NNFI = non-normed fit index.

## Data Availability

Data and code used for analysis, and the results from HRF-GIMME are available online at https://osf.io/ykcr7/ last accessed on 8 October 2021.

## Supplementary Materials

The following are available online at https://www.mdpi.com/article/10.3390/brainsci11111472/s1, Figures S1–S11: effective connectivity maps for all participants, Table S1: Average responses per stimulus, standard deviation, and standard error of the mean during the transfer phase by response type, interval, and stimulus type. Table S2: Results from the three-way repeated measures ANOVA probing the effects of stimulus, response, and interval on instrumental responding during the avoidance-based PIT task. Table S3: Results from the multiple pairwise comparisons post hoc analysis to probe the effects of stimulus type and response compared to the pre-stimulus period.

## References

[1] Balleine B.W., Dickinson A. (1998). Goal-directed instrumental action: Contingency and incentive learning and their cortical substrates. Neuropharmacology.

[2] Rescorla R.A., Solomon R.L. (1967). Two-process learning theory: Relationships between Pavlovian conditioning and instrumental learning. Psychol. Rev..

[3] Meemken M.T., Horstmann A. (2019). Appetitive pavlovian-to-instrumental transfer in participants with normal-weight and obesity. Nutrients.

[4] Watson P., Wiers R.W., Hommel B., De Wit S. (2014). Working for food you don’t desire. Cues interfere with goal-directed food-seeking. Appetite.

[5] Hogarth L., Lam-Cassettari C., Pacitti H., Currah T., Mahlberg J., Hartley L., Moustafa A. (2019). Intact goal-directed control in treatment-seeking drug users indexed by outcome-devaluation and Pavlovian to instrumental transfer: Critique of habit theory. Eur. J. Neurosci..

[6] LeBlanc K.H., Ostlund S.B., Maidment N.T. (2012). Pavlovian-to-instrumental transfer in cocaine seeking rats. Behav. Neurosci..

[7] Hardy L., Mitchell C., Seabrooke T., Hogarth L. (2017). Drug cue reactivity involves hierarchical instrumental learning: Evidence from a biconditional Pavlovian to instrumental transfer task. Psychopharmacology (Berl).

[8] Lewis A.H., Niznikiewicz M.A., Delamater A.R., Delgado M.R. (2013). Avoidance-based human Pavlovian-to-instrumental transfer. Eur. J. Neurosci..

[9] Baker T.B., Piper M.E., McCarthy D.E., Majeskie M.R., Fiore M.C. (2004). Addiction Motivation Reformulated: An Affective Processing Model of Negative Reinforcement. Psychol. Rev..

[10] Cartoni E., Balleine B., Baldassarre G. (2016). Appetitive Pavlovian-instrumental Transfer: A review. Neurosci. Biobehav. Rev..

[11] Corbit L.H., Janak P.H., Balleine B.W. (2007). General and outcome-specific forms of Pavlovian-instrumental transfer: The effect of shifts in motivational state and inactivation of the ventral tegmental area. Eur. J. Neurosci..

[12] Corbit L.H., Balleine B.W. (2005). Double dissociation of basolateral and central amygdala lesions on the general and outcome-specific forms of pavlovian-instrumental transfer. J. Neurosci..

[13] Holmes N.M., Marchand A.R., Coutureau E. (2010). Pavlovian to instrumental transfer: A neurobehavioural perspective. Neurosci. Biobehav. Rev..

[14] Yin H.H., Knowlton B.J. (2006). The role of the basal ganglia in habit formation. Nat. Rev. Neurosci..

[15] Yin H.H., Knowlton B.J., Balleine B.W. (2004). Lesions of dorsolateral striatum preserve outcome expectancy but disrupt habit formation in instrumental learning. Eur. J. Neurosci..

[16] Corbit L.H., Janak P.H. (2007). Inactivation of the lateral but not medial dorsal striatum eliminates the excitatory impact of pavlovian stimuli on instrumental responding. J. Neurosci..

[17] Yin H.H., Ostlund S.B., Knowlton B.J., Balleine B.W. (2005). The role of the dorsomedial striatum in instrumental conditioning. Eur. J. Neurosci..

[18] Bray S., Rangel A., Shimojo S., Balleine B., O’Doherty J.P. (2008). The neural mechanisms underlying the influence of pavlovian cues on human decision making. J. Neurosci..

[19] Fullana M.A., Harrison B.J., Soriano-Mas C., Vervliet B., Cardoner N., Àvila-Parcet A., Radua J. (2016). Neural signatures of human fear conditioning: An updated and extended meta-analysis of fMRI studies. Mol. Psychiatry.

[20] Delgado M.R., Jou R.L., Phelps E.A. (2011). Neural systems underlying aversive conditioning in humans with primary and secondary reinforcers. Front. Neurosci..

[21] Kim H., Shimojo S., O’Doherty J.P. (2006). Is avoiding an aversive outcome rewarding? Neural substrates of avoidance learning in the human brain. PLoS Biol..

[22] Cloutman L.L., Binney R.J., Drakesmith M., Parker G.J.M., Lambon Ralph M.A. (2012). The variation of function across the human insula mirrors its patterns of structural connectivity: Evidence from in vivo probabilistic tractography. Neuroimage.

[23] Calzavara R., Mailly P., Haber S.N. (2007). Relationship between the corticostriatal terminals from areas 9 and 46, and those from area 8A, dorsal and rostral premotor cortex and area 24c: An anatomical substrate for cognition to action. Eur. J. Neurosci..

[24] Duffy K.A., Fisher Z.F., Arizmendi C.A., Molenaar P.C.M., Hopfinger J., Cohen J.R., Beltz A., Lindquist M.A., Hallquist M.N., Gates K. (2021). Detecting task-dependent functional connectivity in GIMME with person-specific hemodynamic response functions. Brain Connect..

[25] Gates K.M., Molenaar P.C.M., Hillary F.G., Slobounov S. (2011). Extended unified SEM approach for modeling event-related fMRI data. Neuroimage.

[26] Handwerker D.A., Ollinger J.M., D’Esposito M. (2004). Variation of BOLD hemodynamic responses across subjects and brain regions and their effects on statistical analyses. Neuroimage.

[27] Finn E.S., Shen X., Scheinost D., Rosenberg M.D., Huang J., Chun M.M., Papademetris X., Constable R.T. (2015). Functional connectome fingerprinting: Identifying individuals using patterns of brain connectivity. Nat. Neurosci..

[28] Laumann T.O., Gordon E.M., Adeyemo B., Snyder A.Z., Joo S.J., Chen M.Y., Gilmore A.W., McDermott K.B., Nelson S.M., Dosenbach N.U.F. (2015). Functional System and Areal Organization of a Highly Sampled Individual Human Brain. Neuron.

[29] Molenaar P.C.M. (2004). A Manifesto on Psychology as Idiographic Science: Bringing the Person Back Into Scientific Psychology, This Time Forever. Measurement.

[30] Molenaar P.C.M., Campbell C.G. (2009). The new person-specific paradigm in psychology. Curr. Dir. Psychol. Sci..

[31] Gates K.M., Peter C.M. (2012). Molenaar Group search algorithm recovers effective connectivity maps for individuals in homogeneous and heterogeneous samples. Neuroimage.

[32] Smith S.M., Miller K.L., Salimi-Khorshidi G., Webster M., Beckmann C.F., Nichols T.E., Ramsey J.D., Woolrich M.W. (2011). Network modelling methods for FMRI. Neuroimage.

[33] Friston K.J., Buechel C., Fink G.R., Morris J., Rolls E., Dolan R.J. (1997). Psychophysiological and modulatory interactions in neuroimaging. Neuroimage.

[34] Sarty G.E. (2007). Computing Brain Activity Maps from fMRI Time-Series Images.

[35] Friston K.J., Harrison L., Penny W. (2003). Dynamic causal modelling. Neuroimage.

[36] Nadler N., Delgado M.R., Delamater A.R. (2011). Pavlovian to Instrumental Tansfer of Control in a Human Learning Task. Emotion.

[37] Molenaar P.C.M., Nesselroade J.R. (1998). A Comparison of pseudo-Maximum Likelihood and Asymptotically Distribution-Free dynamic factor analysis parameter estimation in fitting covariance-structure models to block-Toeplitz matrices representing single-subject multivariate time-series. Multivar. Behav. Res..

[38] Holm S. (1979). A Simple Sequentially Rejective Multiple Test Procedure. Scand. J. Stat..

[39] Cox R.W. (1996). AFNI: Software for analysis and visualization of functional magnetic resonance neuroimages. Comput. Biomed. Res..

[40] Granger C.J.W. (1969). Investigating Causal Relations by Econometric Models and Cross-spectral Methods. Econometrica.

[41] Lane S.T., Gates K.M., Fisher Z., Arizmendi C., Molenaar P.C.M., Hallquist M., Pike H., Henry T., Duffy K., Luo L. GIMME: Group Iterative Multiple Model Estimation 2020, R Package Version 0.7-3. https://CRAN.R-project.org.

[42] Gates K.M., Molenaar P.C.M., Hillary F.G., Ram N., Rovine M.J. (2010). Automatic search for fMRI connectivity mapping: An alternative to Granger causality testing using formal equivalences among SEM path modeling, VAR, and unified SEM. Neuroimage.

[43] Beltz A.M., Gates K.M. (2017). Network Mapping with GIMME. Multivar. Behav. Res..

[44] Hillary F.G., Medaglia J.D., Gates K., Molenaar P.C., Slocomb J., Peechatka A., Good D.C. (2011). Examining working memory task acquisition in a disrupted neural network. Brain.

[45] Goutte C., Nielsen F., Hansen K. (2000). Modeling the hemodynamic response in fMRI using smooth FIR filters. IEEE Trans. Med. Imaging.

[46] Lindquist M.A., Meng Loh J., Atlas L.Y., Wager T.D. (2009). Modeling the hemodynamic response function in fMRI: Efficiency, bias and mis-modeling. Neuroimage.

[47] Sörbom D. (1989). Model modification. Psychometrika.

[48] Klein M., Rilling M. (1971). Effects of Response-shock Interval and Shock Intensity on Free-operant Avoidance Responding in the Pigeon. J. Exp. Anal. Behav..

[49] Sidman M. (1962). Classical Avoidance Without a Warning Stimulus. J. Exp. Anal. Behav..

[50] Ulrich R.E., Holz W.C., Azrin N.H. (1964). Stimulus Control of Avoidance Behavior. J. Exp. Anal. Behav..

[51] Krypotos A.M., Engelhard I.M. (2020). Pavlovian-to-instrumental transfer in subclinical obsessive–compulsive disorder. J. Exp. Psychopathol..

[52] Talmi D., Seymour B., Dayan P., Dolan R.J. (2008). Human pavlovian-instrumental transfer. J. Neurosci..

[53] Prévost C., Liljeholm M., Tyszka J.M., O’Doherty J.P. (2012). Neural correlates of specific and general pavlovian-to-instrumental transfer within Human Amygdalar Subregions: A high-resolution fMRI study. J. Neurosci..

[54] de Wit S., Watson P., Harsay H.A., Cohen M.X., van de Vijver I., Ridderinkhof K.R. (2012). Corticostriatal connectivity underlies individual differences in the balance between habitual and goal-directed action control. J. Neurosci..

[55] Draganski B., Kherif F., Klöppel S., Cook P.A., Alexander D.C., Parker G.J.M., Deichmann R., Ashburner J., Frackowiak R.S.J. (2008). Evidence for segregated and integrative connectivity patterns in the human basal ganglia. J. Neurosci..

[56] Lehéricy S., Ducros M., Van De Moortele P.F., Francois C., Thivard L., Poupon C., Swindale N., Ugurbil K., Kim D.S. (2004). Diffusion Tensor Fiber Tracking Shows Distinct Corticostriatal Circuits in Humans. Ann. Neurol..

[57] van Steenbergen H., Watson P., Wiers R.W., Hommel B., de Wit S. (2017). Dissociable corticostriatal circuits underlie goal-directed vs. cue-elicited habitual food seeking after satiation: Evidence from a multimodal MRI study. Eur. J. Neurosci..

[58] Krzyzak A.T., Olejniczak Z. (2015). Improving the accuracy of PGSE DTI experiments using the spatial distribution of b matrix. Magn. Reson. Imaging.

[59] Kłodowski K., Krzyzak A.T. (2016). Innovative anisotropic phantoms for calibration of diffusion tensor imaging sequences. Magn. Reson. Imaging.

[60] Borkowski K., Krzyżak A.T. (2018). Analysis and correction of errors in DTI-based tractography due to diffusion gradient inhomogeneity. J. Magn. Reson..

[61] Borkowski K., Krzyżak A.T. (2018). The generalized Stejskal-Tanner equation for non-uniform magnetic field gradients. J. Magn. Reson..

[62] Mechias M.L., Etkin A., Kalisch R. (2010). A meta-analysis of instructed fear studies: Implications for conscious appraisal of threat. Neuroimage.

[63] Gerlicher A., Kindt M. (2020). A Review on Aversive Pavlovian-to-Instrumental Transfer in Humans.

