# Supplementary material for: Effective Connectivity during an Avoidance-Based Pavlovian-to-Instrumental Transfer Task
Source: Brain Sci. 2021 Nov 6;11(11):1472. doi: 10.3390/brainsci11111472 (PMC8615846; doi:10.3390/brainsci11111472)
Supplement: Supplementary file 1 [file brainsci-11-01472-s001.zip › brainsci-1366500-supplementary.pdf]

Supplementary Materials

Supplementary Tables

Table S1: Average responses per stimulus, standard deviation, and standard error of the mean during the transfer phase by response type (R1 or R2), interval (pre-stimulus or stimulus) and stimulus type (CS1 – CS5).

Response	Interval	Stimulus	Response per Stimulus	SD	SEM
R1	Pre	CS1	0.99	2.38	0.72
R1	Pre	CS2	2.02	4.68	1.41
R1	Pre	CS3	0.72	1.55	0.47
R1	Pre	CS4_neu	1.05	2.59	0.78
R1	Pre	CS5_mal	1.48	3.42	1.03
R1	Stim	CS1	11.86	7.73	2.33
R1	Stim	CS2	0.55	0.65	0.20
R1	Stim	CS3	3.66	6.09	1.83
R1	Stim	CS4_neu	0.05	0.13	0.04
R1	Stim	CS5_mal	0.06	0.14	0.04
R2	Pre	CS1	0.63	1.41	0.42
R2	Pre	CS2	1.17	2.61	0.79
R2	Pre	CS3	1.31	3.17	0.96
R2	Pre	CS4_neu	1.83	5.12	1.54
R2	Pre	CS5_mal	1.16	2.61	0.79
R2	Stim	CS1	0.29	0.44	0.13
R2	Stim	CS2	11.61	8.04	2.42
R2	Stim	CS3	1.43	1.95	0.59
R2	Stim	CS4_neu	0.08	0.20	0.06
R2	Stim	CS5_mal	0.05	0.10	0.03

Note. SD = standard deviation, SEM = standard error of the mean.

Table S2: Results from the 3-way repeated measures ANOVA probing the effects of stimulus, response, and interval on instrumental responding during the avoidance-based PIT task.

Effect	F (df_n, df_d)	η^2_g
Stimulus	15.18 (2, 20.01)*	0.126
Response	2.11 (1, 10)	0.002
Interval	2.19 (1, 10)	0.058
Stimulus x Response	12.19 (1.67, 16.73)*	0.205
Stimulus x Interval	19.42 (1.90, 19.01)*	0.138
Response x Interval	1.64 (1, 10)	0.001
Stimulus x Response x Interval	15.43 (1.89, 18.86)*	0.221

Note. * $p < 0.05$, η^2_g = generalized eta-squared.

Table S3: Results from the multiple pairwise comparisons *post hoc* analysis to probe the effects of stimulus type and response compared to the pre-stimulus period.

Stimulus	Response	*t*-values: Pre vs Stim	*p* adjusted
CS1	R1	-4.00	0.003*
	R2	0.76	0.465
CS2	R1	1.03	0.325
	R2	-3.81	0.003*
CS3	R1	-1.46	0.174
	R2	-0.10	0.922
CS4_neu	R1	1.34	0.209
	R2	1.18	0.266
CS5_mal	R1	1.44	0.180
	R2	1.45	0.178

Note. * $p < 0.005$, CS = conditioned stimulus, R = response. Pre = pre-stimulus presentation, Stim = stimulus presentation. P-values were adjusted using the Bonferroni corrected alpha level of 0.005 (0.05/10).

Supplementary Figures

Supplementary figures S1 – S11 display connectivity maps retained for all participants in the study (*N* = 11). Red lines indicate a positive connection, blue lines indicate a negative connection, solid lines indicate contemporaneous connections, dashed lines indicate a lagged connection of the first order. The thickness of the lines indicates the magnitude of the connections, such that thicker lines indicate a stronger magnitude of connectivity. Note that variables specific and general indicate the direct effect of the task on the other ROIs. Note that variables Lputamen_by_specific and Lputamen_by_general indicate bilinear effects of the contemporaneous relations among the left putamen and our selected *a priori* ROIs depending on the input time series $u_{i,t}$. In other words, the relation between left putamen and our selected *a priori* ROIs differ according to whether participants were on or off task.

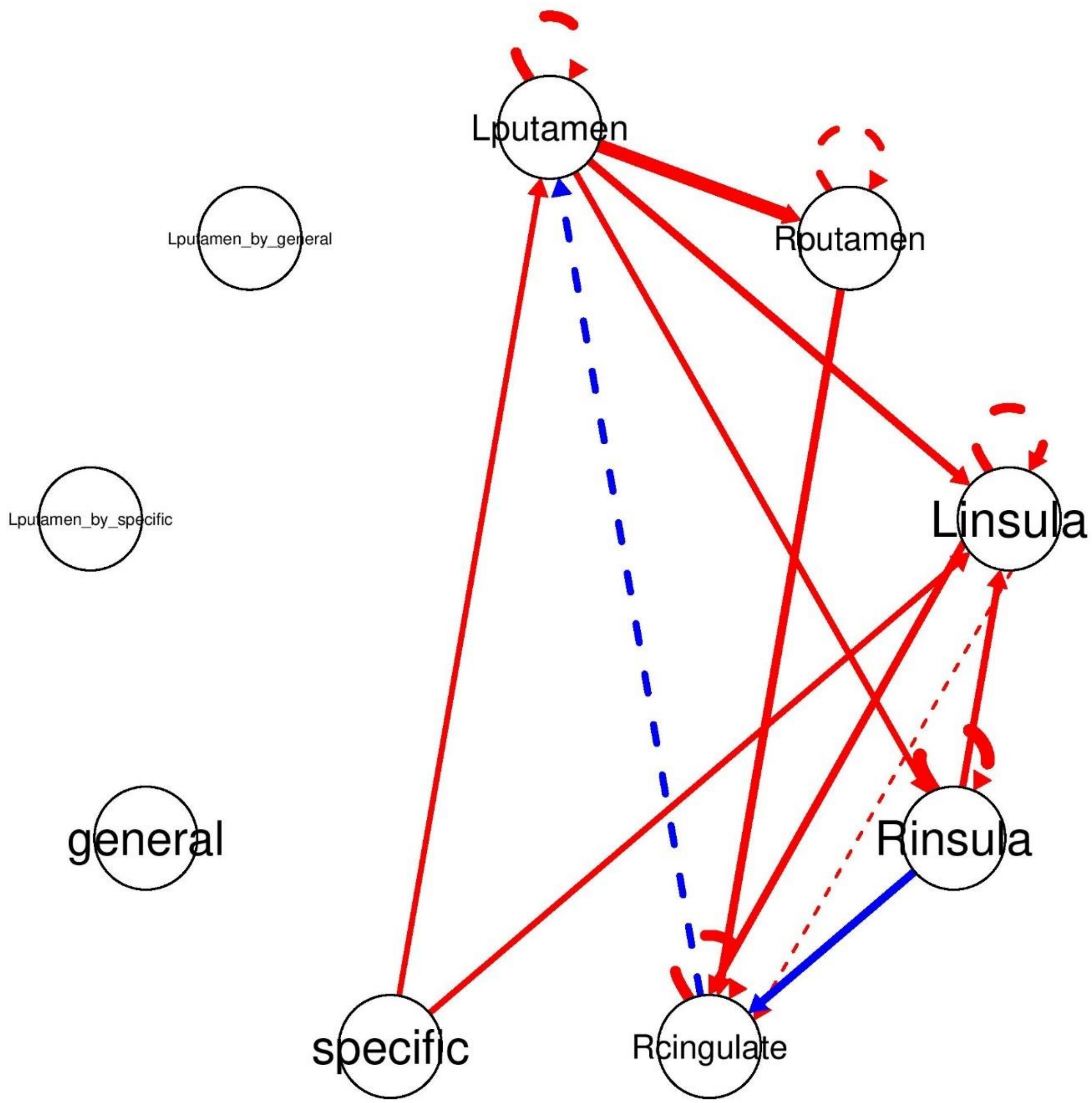

Figure S1. Participant 1

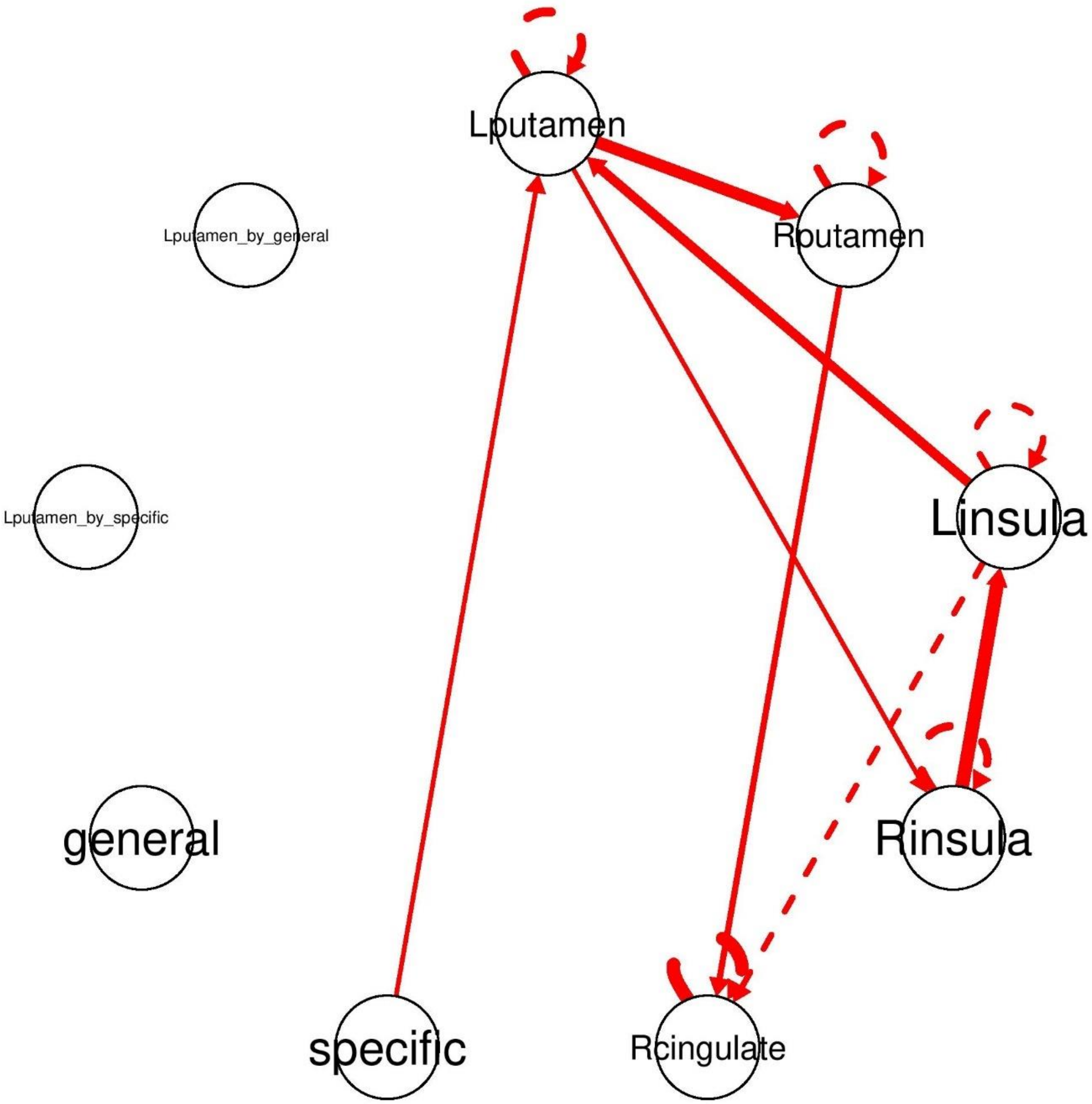

Figure S2. Participant 2

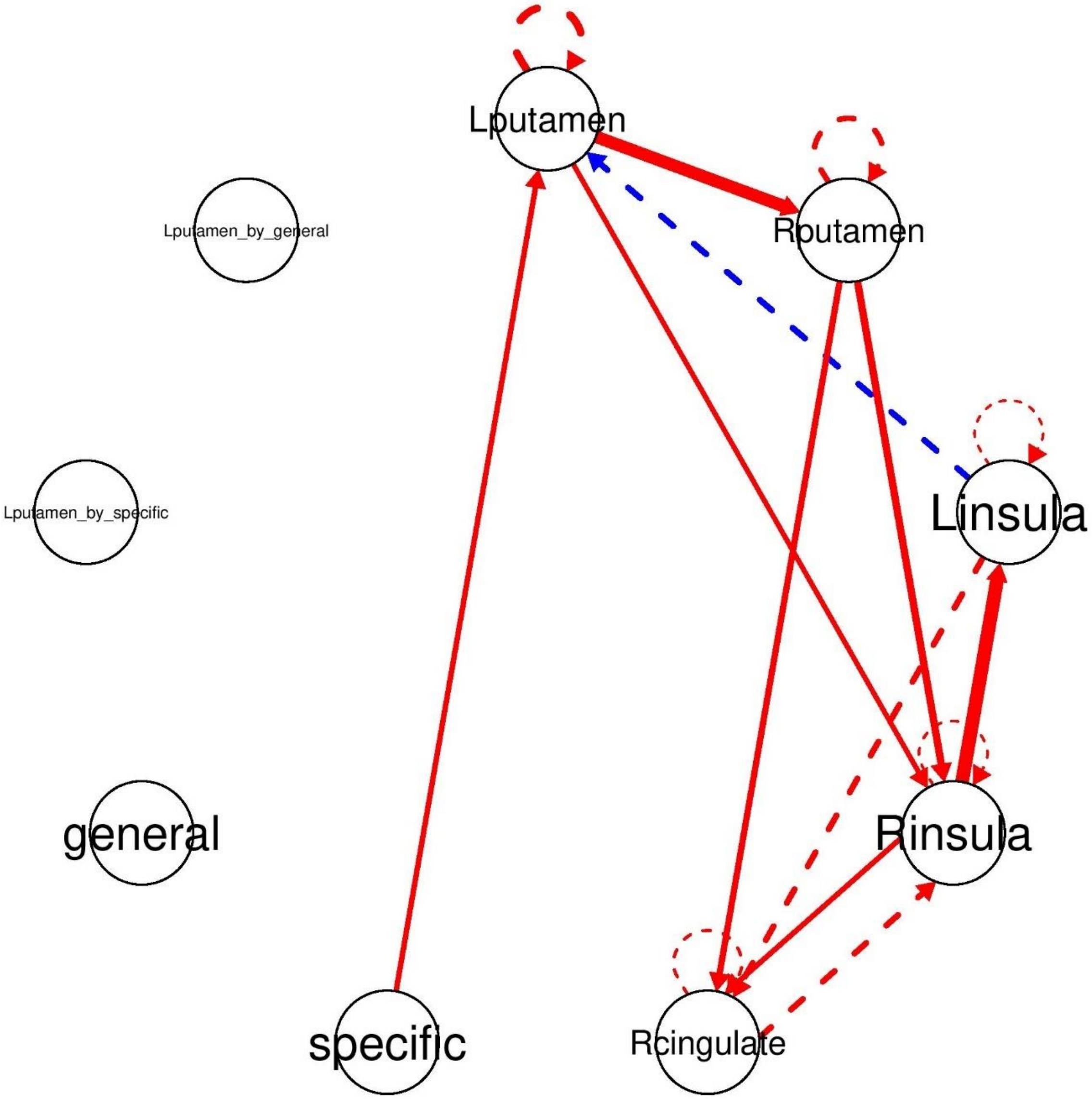

Figure S3. Participant 3

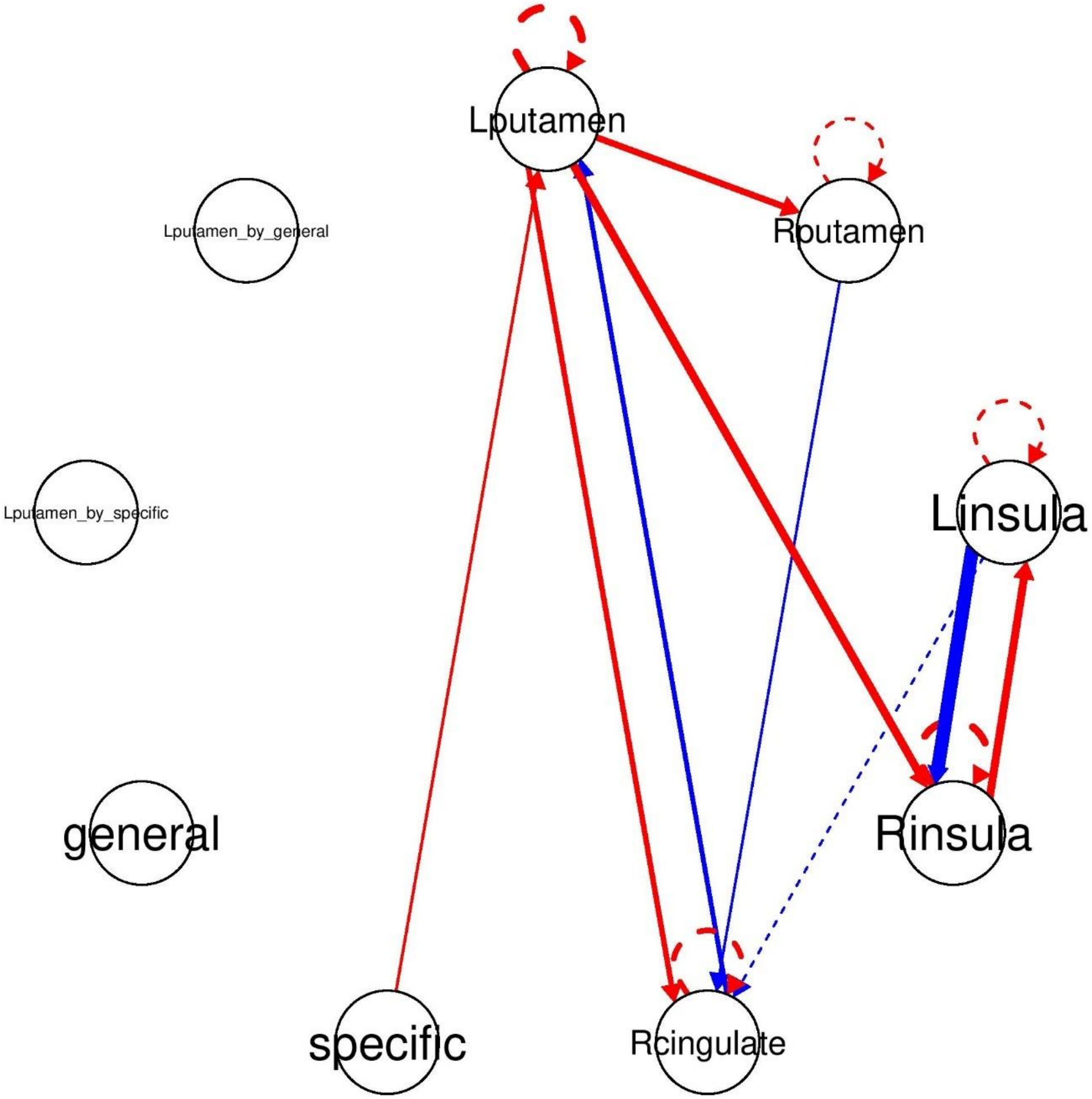

Figure S4. Participant 4

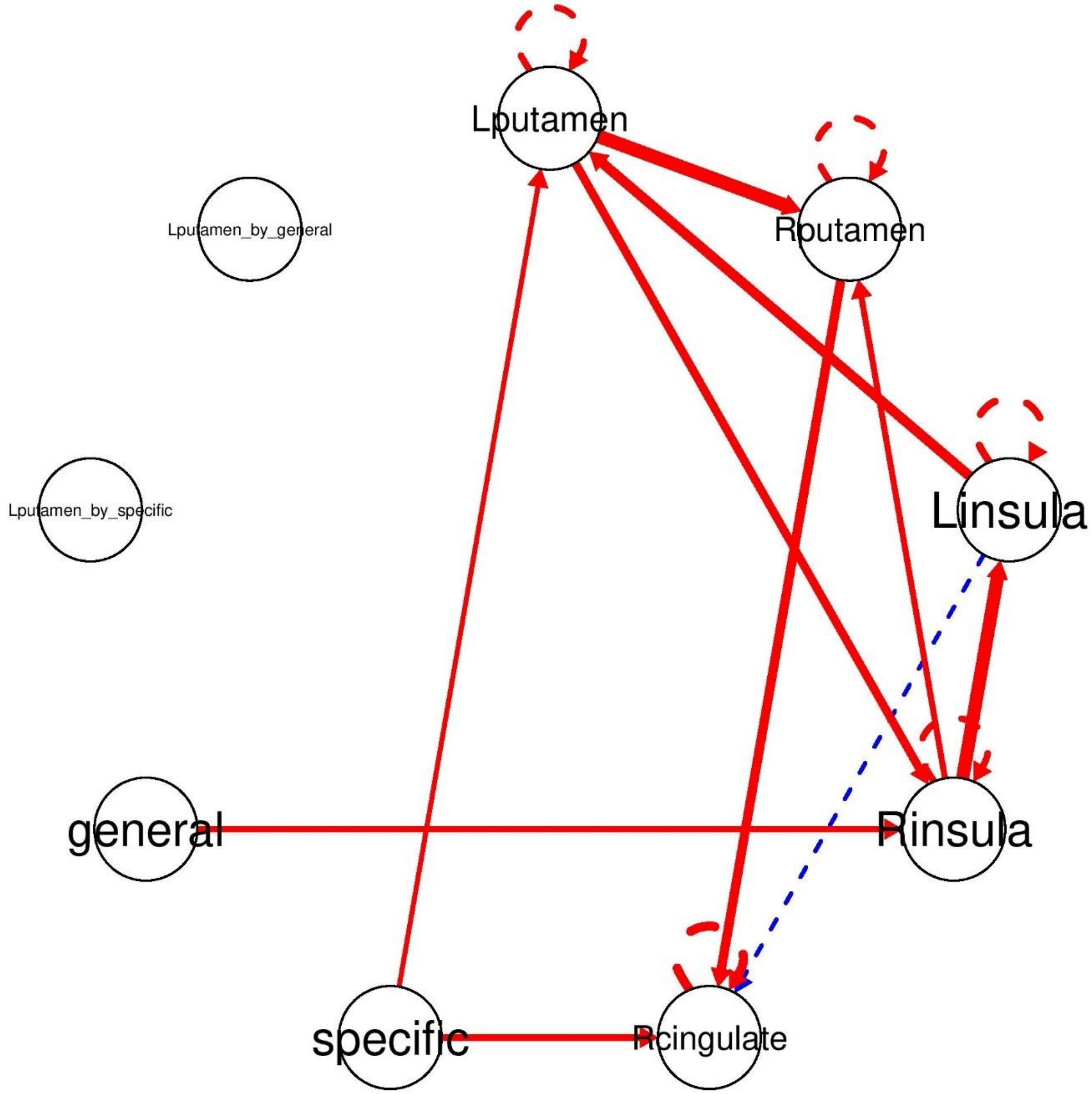

Figure S5. Participant 5

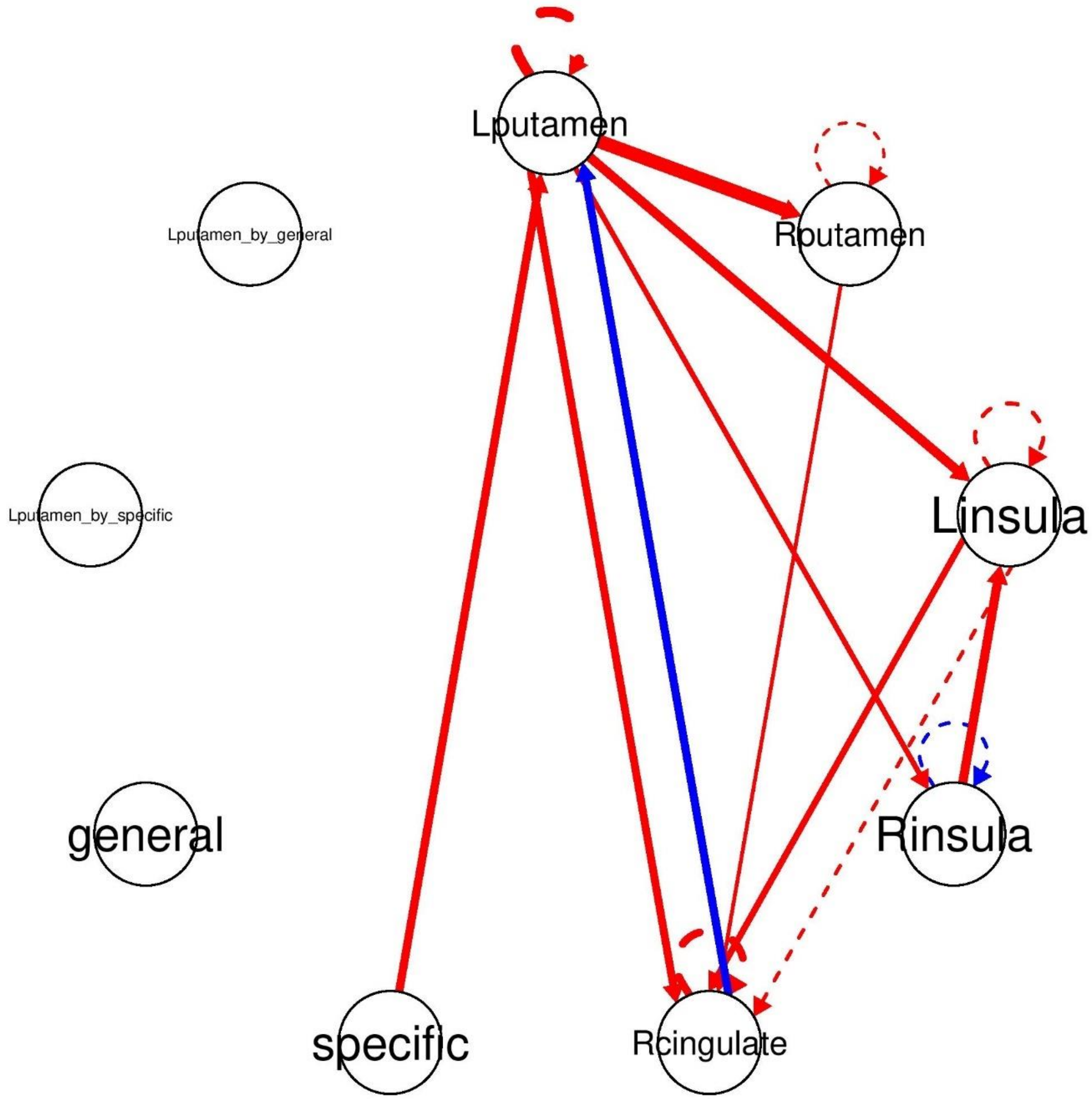

Figure S6. Participant 6

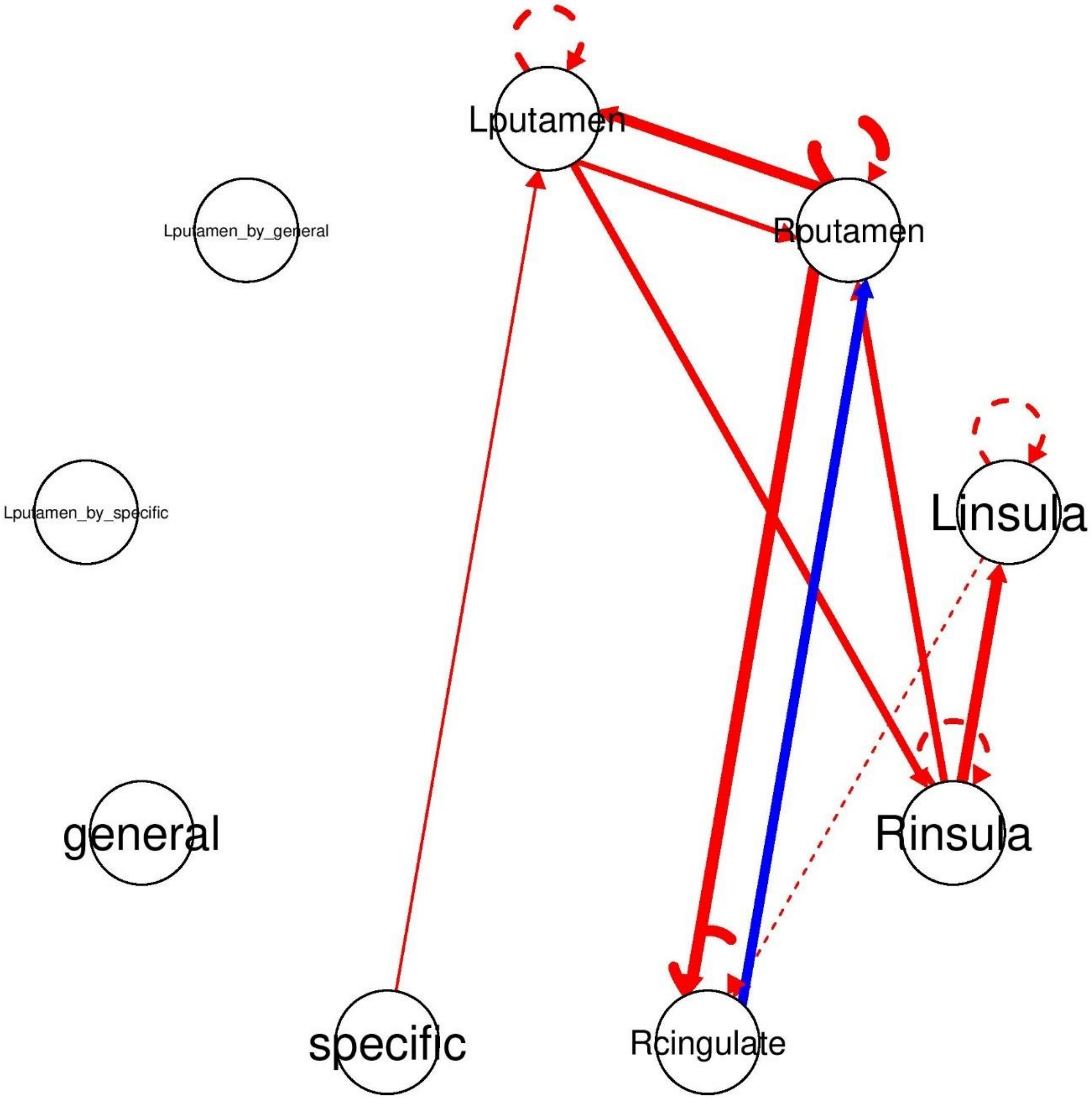

Figure S7. Participant 7

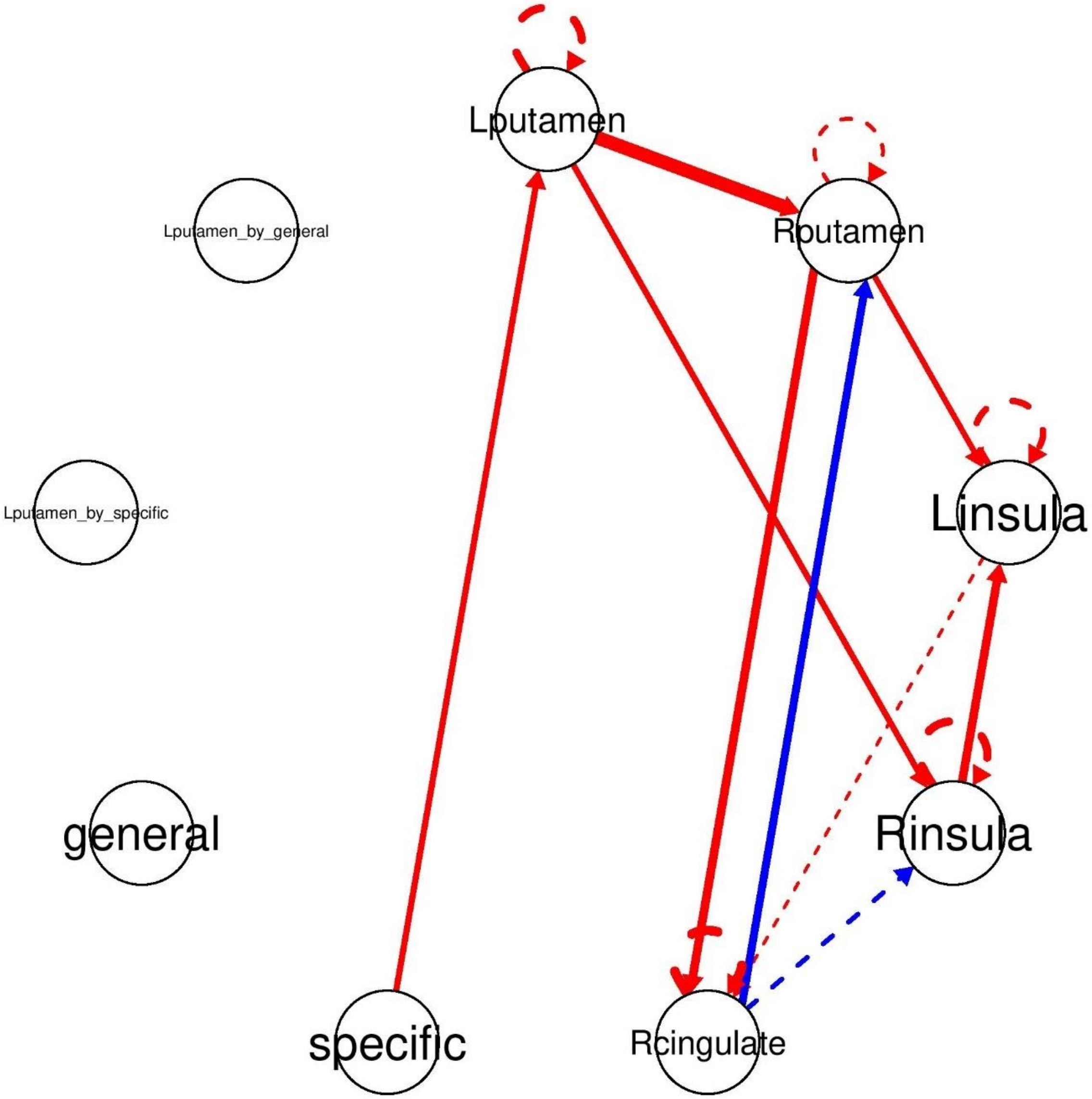

Figure S8. Participant 8

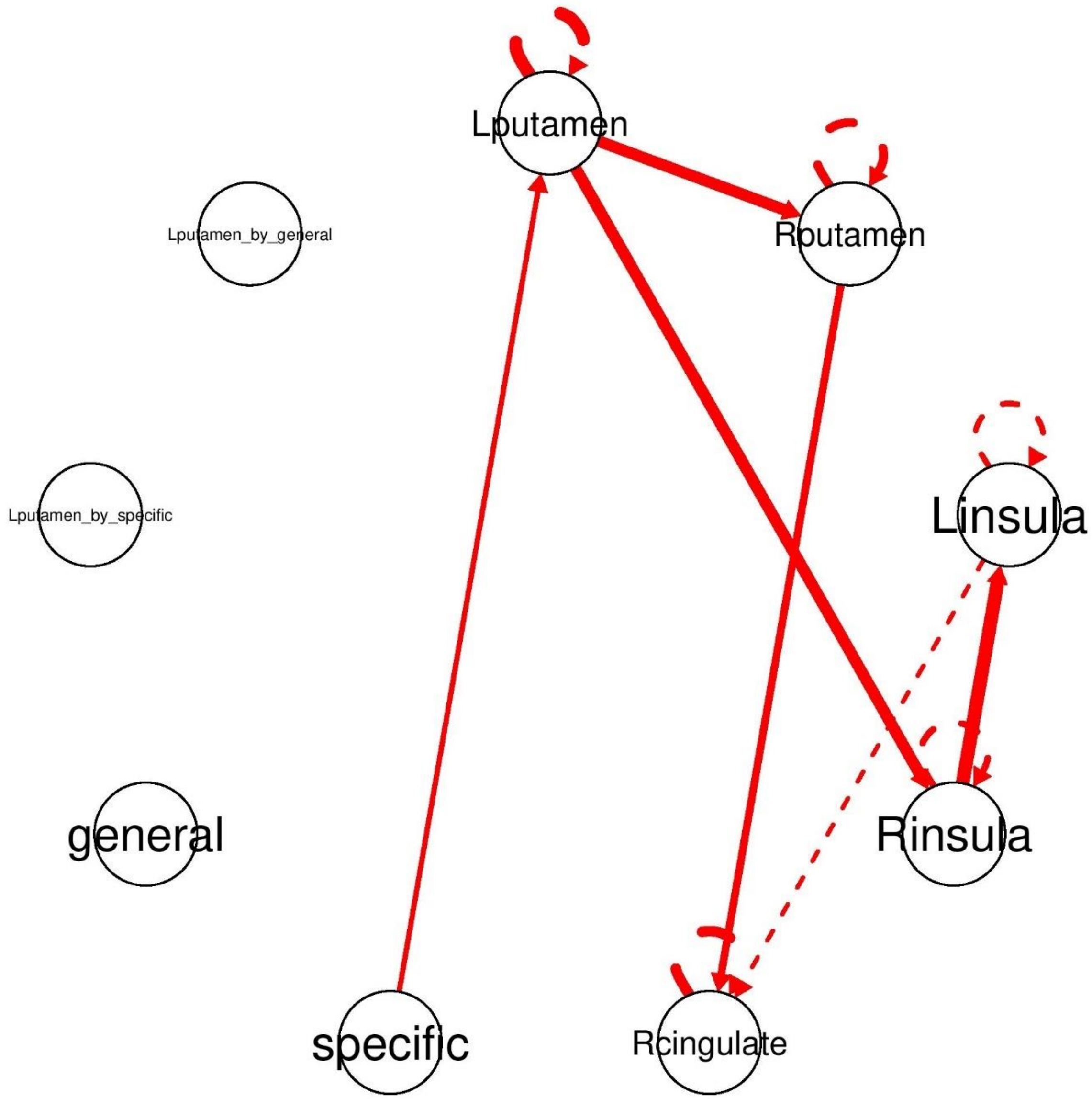

Figure S9. Participant 9

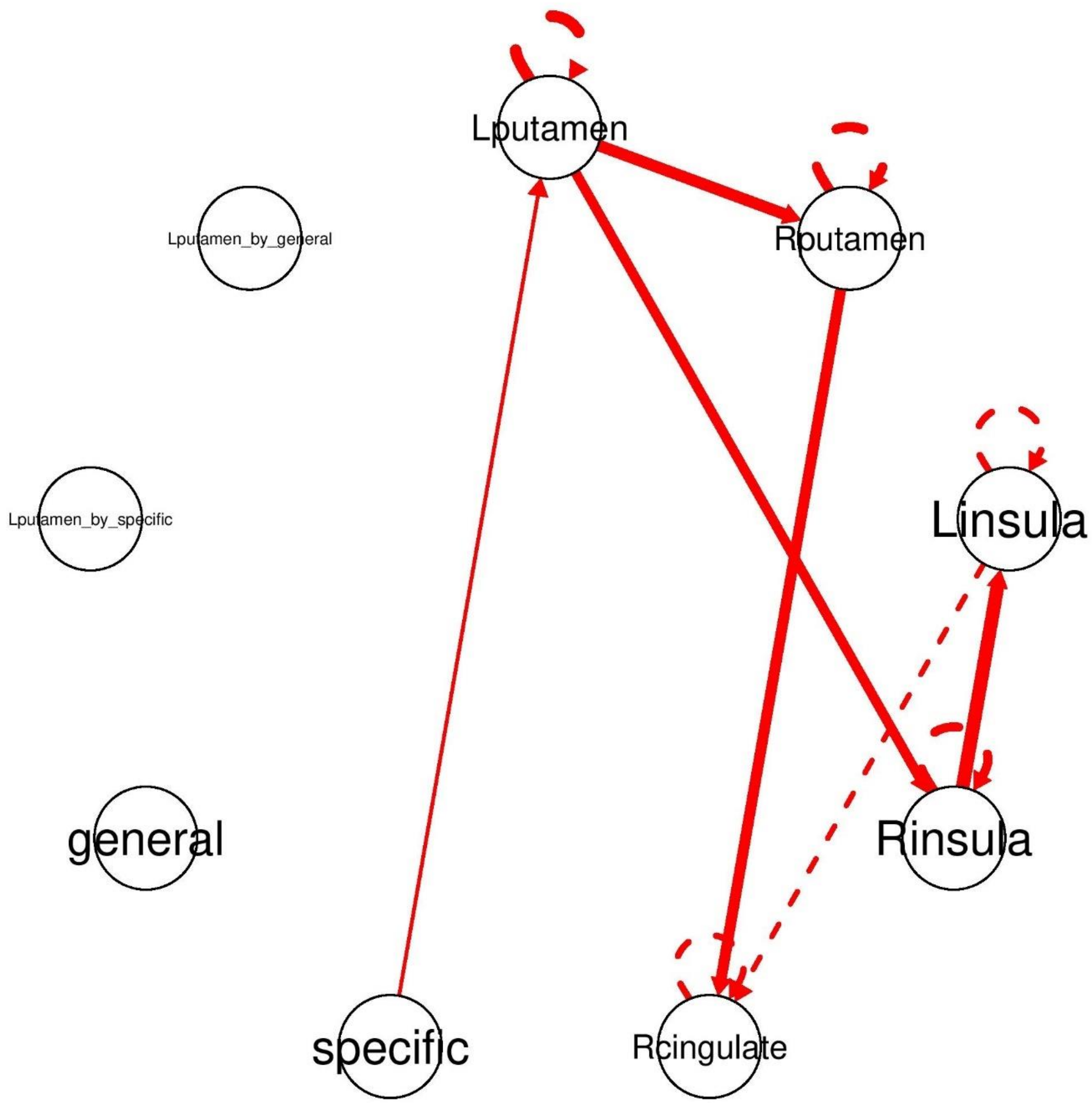

Figure S10. Participant 10

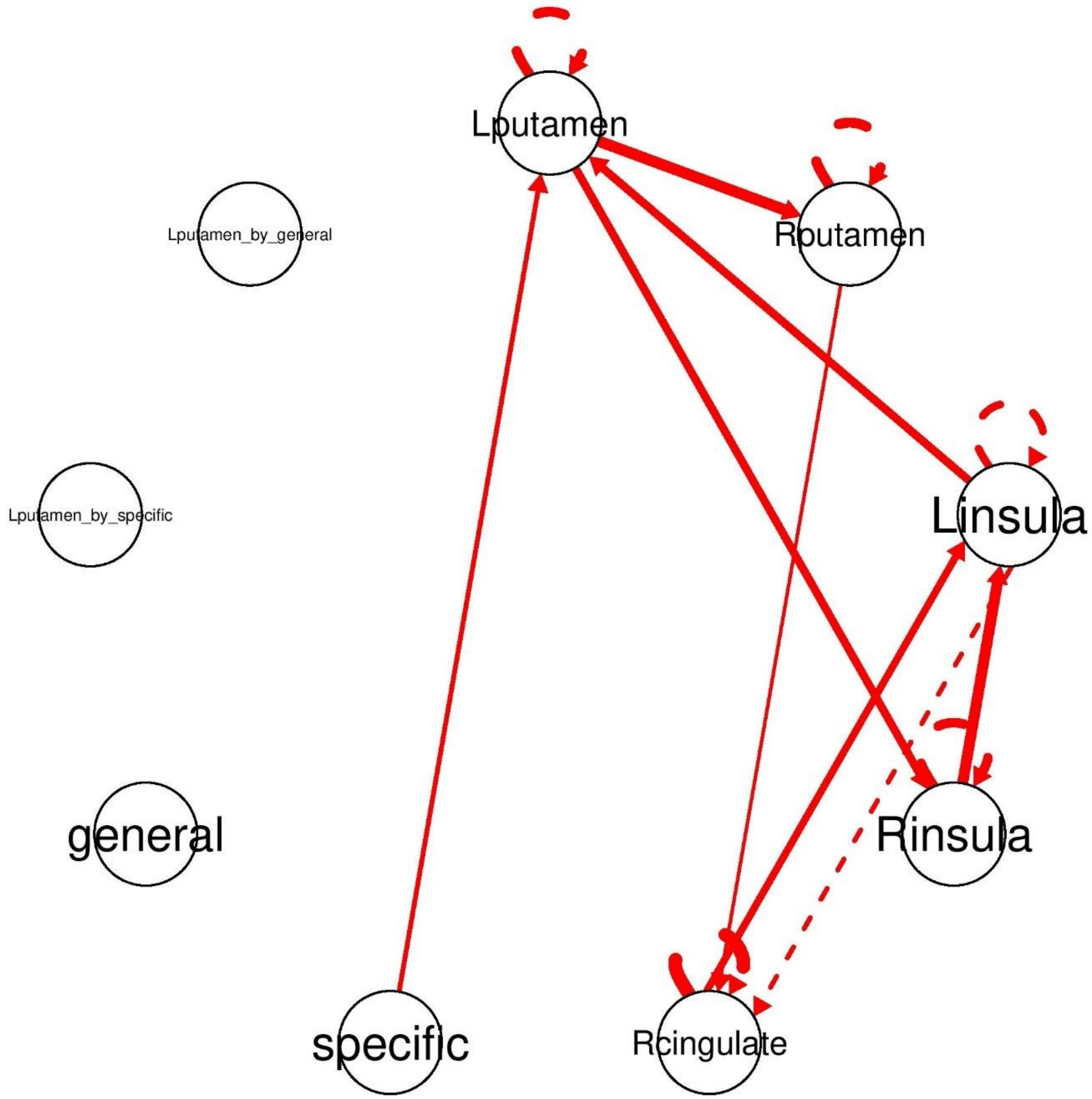

Figure S11. Participant 11
